# Long-chain acyl-CoA synthetases in female reproductive diseases: isoform-specific regulation of lipid metabolism, ferroptosis, and inflammatory signaling

**DOI:** 10.3389/fendo.2026.1967984

**Published:** 2026-10-28

**Authors:** Shanyuan Jin, Lin Guo, Zishuo Wu, Zirong Wang, Runyuan Liu, Ying Kong, Hongshuo Zhang

**Affiliations:** 1 Department of Biochemistry and Molecular Biology, College of Basic Medical Sciences, Dalian Medical University, Dalian, China; 2 Advanced Institute for Medical Sciences, Dalian Medical University, Dalian, China; 3 Department of Otorhinolaryngology, the Second Hospital of Dalian Medical University, Dalian, China; 4 School of Basic Medical Sciences, Dalian University of Technology, Dalian, China

**Keywords:** female reproductive diseases, long-chain acyl-CoA synthetase, lipid metabolic reprogramming, ferroptosis, inflammatory signaling.

## Abstract

Female reproductive diseases (FRDs) substantially affect women’s health and fertility, with lipid metabolic reprogramming emerging as a shared pathological feature. Long-chain acyl-CoA synthetases (ACSLs) activate long-chain fatty acids and direct lipid flux toward β-oxidation, lipid synthesis, and phospholipid remodeling, thereby influencing oxidative stress, ferroptosis, and inflammatory signaling. Unlike previous reviews centered on broad ACSL biology, selected isoforms, or pathway-based ferroptosis mechanisms, this review adopts a disease-spanning, isoform-specific framework. We compare ACSL1, ACSL3, ACSL4, ACSL5, and ACSL6 across cervical, ovarian, and endometrial cancers, polycystic ovary syndrome, endometriosis, and preeclampsia. By integrating isoform-specific substrate preference, subcellular localization, and lipid-channeling functions, we examine how individual ACSLs differentially regulate fatty acid oxidation, lipid synthesis and phospholipid remodeling, ferroptosis sensitivity, and inflammatory signaling across reproductive disease contexts. We further distinguish disease-specific experimental evidence from bioinformatic associations and mechanistic extrapolations from other disease models. Current evidence most consistently supports a disease-relevant role for ACSL4 in ferroptosis regulation, whereas ACSL1, ACSL3, ACSL5, and ACSL6 show greater context dependence. This integrated framework identifies shared metabolic mechanisms, disease-specific differences, and priorities for cell-resolved validation and future isoform-selective therapeutic development.

## 1 Introduction

The health of the female reproductive system profoundly impacts individual quality of life and sustainable population development (1). However, a series of complex female reproductive diseases (FRDs) severely affect the physiological functions, psychological states, and fertility of hundreds of millions of women worldwide, constituting a major public health challenge. These disorders often exhibit multifactorial etiologies, intertwined mechanisms, and clinical heterogeneity, with strong interrelationships that complicate diagnosis and treatment. Despite differences in clinical manifestations and target organs among various FRDs, growing evidence suggests they may share core pathological mechanisms, such as lipid metabolism reprogramming, oxidative stress, ferroptosis, and cellular energy metabolism (2). Among these, lipid metabolism abnormalities have increasingly been identified as a key common pathway. Lipid metabolic reprogramming contributes to disease onset and progression through multiple mechanisms, including regulation of cellular signaling, membrane stability, and inflammatory responses (3). Therefore, re-examining FRDs from a metabolic perspective—particularly by focusing on key regulatory molecules in lipid metabolism such as the ACSLs—holds promise for revealing their common pathogenic mechanisms and developing novel intervention strategies.

Long-chain acyl-CoA synthetases (ACSLs) regulate physiological and pathological processes in the female reproductive system by modulating fatty acid activation, lipid synthesis, and lipid signaling pathways (4). Unlike short- and medium-chain acyl-CoA synthetases, ACSLs preferentially activate long-chain fatty acids, typically containing 12–20 carbon atoms, to form fatty acyl-CoAs, thereby supporting fatty acid oxidation, lipid synthesis, and membrane remodeling. Among ACSL isoforms, ACSL4 provides a particularly relevant link between lipid activation and hormone-dependent biology. Early functional studies demonstrated that suppression of ACSL4 impairs hormone-stimulated steroidogenesis, supporting a role for ACSL4-dependent arachidonic acid metabolism in steroidogenic signaling and StAR-associated cholesterol transport (5). Subsequent studies further linked ACSL4 dysregulation to enhanced proliferation, migration, invasion, and therapeutic resistance in hormone-responsive and hormone-refractory tumor models (6). Extensive research indicates that abnormal ACSL expression and function are closely associated with lipid metabolism reprogramming in various FRDs, including endometrial cancer (EC) and polyendocrine metabolic ovarian syndrome (PMOS), previously known as polycystic ovary syndrome (PCOS) (7–9). Therefore, this review aims to systematically examine the isoform-specific roles and regulatory mechanisms of ACSLs across FRDs, thereby clarifying their shared and disease-specific metabolic mechanisms and identifying priorities for future mechanistic and translational studies. ACSLs catalyze the ATP-dependent two-step activation of long-chain fatty acids at membrane-associated sites, including the endoplasmic reticulum and the outer mitochondrial membrane, generating long-chain acyl-CoAs that can subsequently enter fatty acid oxidation (FAO) or lipid biosynthetic pathways (10). In mammals, although ACSLs share overlapping catalytic functions, they exhibit distinct patterns of tissue distribution, subcellular localization, and substrate preference (11)(**Table 1**). Such specificity suggests that each ACSL isoform is precisely positioned to channel fatty acids into specific metabolic pathways, thereby acting as a “gatekeeper” of lipid flux. Accumulating evidence indicates that dysregulation of ACSL expression and activity is closely associated with a spectrum of metabolic disorders, including hepatic steatosis, insulin resistance, atherosclerosis, neurological diseases, and certain types of cancer (14), establishing ACSLs as central regulators of cellular energy homeostasis and lipid signaling.

**Table 1:** Characteristics of the ACSLs.

ACSL	Subcellular distribution	Function	Tissue expression (From High to Low)	Chromosome localization	Size (number of amino acids)	Molecular mass (Da)
ACSL1	Outer mitochondrial membrane, endoplasmic reticulum, peroxisomal membrane, cytoplasmic lipid droplet	Mitochondrial β-oxidation: Occurs at the mitochondrial outer membrane, supplies substrates for CPT1. Peroxisomal β-oxidation: Provides substrates for this process. Involved in triglyceride synthesis in the liver and adipose tissue.	Heart, skeletal muscle, brown adipose tissue, white adipose tissue, liver, and kidney – expressed most abundantly in tissues with high energy demands.	4q35.1	698	77,943
ACSL3	Endoplasmic reticulum, cytoplasmic lipid droplet, nuclear envelope	Lipid droplet formation: Highly enriched at the endoplasmic reticulum and nascent lipid droplets. Membrane phospholipid synthesis: Provides acyl-CoA substrates for phosphatidic acid synthesis. Signal transduction: May participate in nuclear signaling regulation through the generation of acyl-CoA.	Liver, lung, brain, mammary gland, adrenal gland, and prostate — expressed at higher levels in tissues with active lipid synthesis.	2q36.1	720	80,420
ACSL4	Endoplasmic reticulum (ER), including mitochondria-associated ER membranes (MAMs); peroxisomes; plasma membrane	Steroidogenesis; preferential activation of PUFAs; phospholipid remodeling and regulation of ferroptosis sensitivity; lipid mediator synthesis; association with tumor aggressiveness in hormone-dependent and other solid tumors (6, 12, 13).	Brain, adrenal gland, testis, ovary, macrophages, and liver — abundantly present in steroidogenic tissues and inflammatory cells.	Xq23	711	79,188
ACSL5	Outer mitochondrial membrane, endoplasmic reticulum	Intestine: In intestinal epithelial cells, activates dietary fatty acids, directs them toward triglyceride synthesis, and participates in chylomicron formation. Liver and adipose tissue: Involved in both fatty acid oxidation and triglyceride synthesis.	Small intestine, liver, white adipose tissue, stomach, and testis — highest expression is observed in the intestinal tract.	10q25.2	683	75,991
ACSL6	Plasma membrane, endoplasmic reticulum	Brain lipid metabolism: Preferentially activates long-chain polyunsaturated fatty acids and incorporates them into brain phospholipids. Vesicular trafficking: May play a role in synaptic vesicle transport and fusion.	Brain, neurons, retina, heart, and skeletal muscle — predominant in the nervous system.	5q31	697	77,752

This table summarizes the major characteristics of ACSLs, including their subcellular distribution, biological functions, tissue expression patterns, chromosomal localization, protein size, and molecular mass. ACSL isoforms show distinct intracellular localizations and tissue expression profiles, suggesting that different ACSL members may participate in lipid metabolism through isoform-specific mechanisms.

In recent years, ACSLs have been recognized not only for their metabolic roles but also for directly regulating cellular signaling and fate. This functional expansion centers on fatty acyl-CoAs, which act as both a metabolic intermediate and a signaling/modification substrate. Ferroptosis is an iron-dependent form of regulated cell death driven by the accumulation of lipid peroxides in cellular membranes (15). ACSL-mediated fatty acid activation and phospholipid remodeling can influence ferroptosis sensitivity by determining the availability of oxidizable lipid substrates. In particular, ACSL4 preferentially activates PUFAs, especially AA and AdA, facilitating their incorporation into membrane phospholipids and thereby increasing susceptibility to lipid peroxidation and ferroptosis (16, 17). This lipid-remodeling function renders ACSL4 a major determinant of ferroptosis sensitivity, as established by Doll et al. (17). ACSL-mediated lipid remodeling also intersects with inflammatory signaling. ACSL4-dependent AA metabolism can influence the availability of substrates for eicosanoid synthesis, including prostaglandins and leukotrienes, thereby providing a biochemical link between lipid metabolism, ferroptosis, and inflammatory signaling (18). In addition, specific ACSL isoforms, such as ACSL1 and ACSL3, have been implicated in the regulation of gene expression and stress responses through enzyme activity-independent functions, acting as signaling scaffolds or transcriptional regulators (19). Therefore, ACSLs can be considered core molecular nodes linking cellular metabolic states with a broad range of biological processes, including cell death, signal transduction, and gene expression, and their dysregulation may be closely associated with multiple ferroptosis-related diseases. The mechanisms by which ACSLs regulate ferroptosis through lipid remodeling are summarized schematically (**Figure 1**).

**Figure 1:**
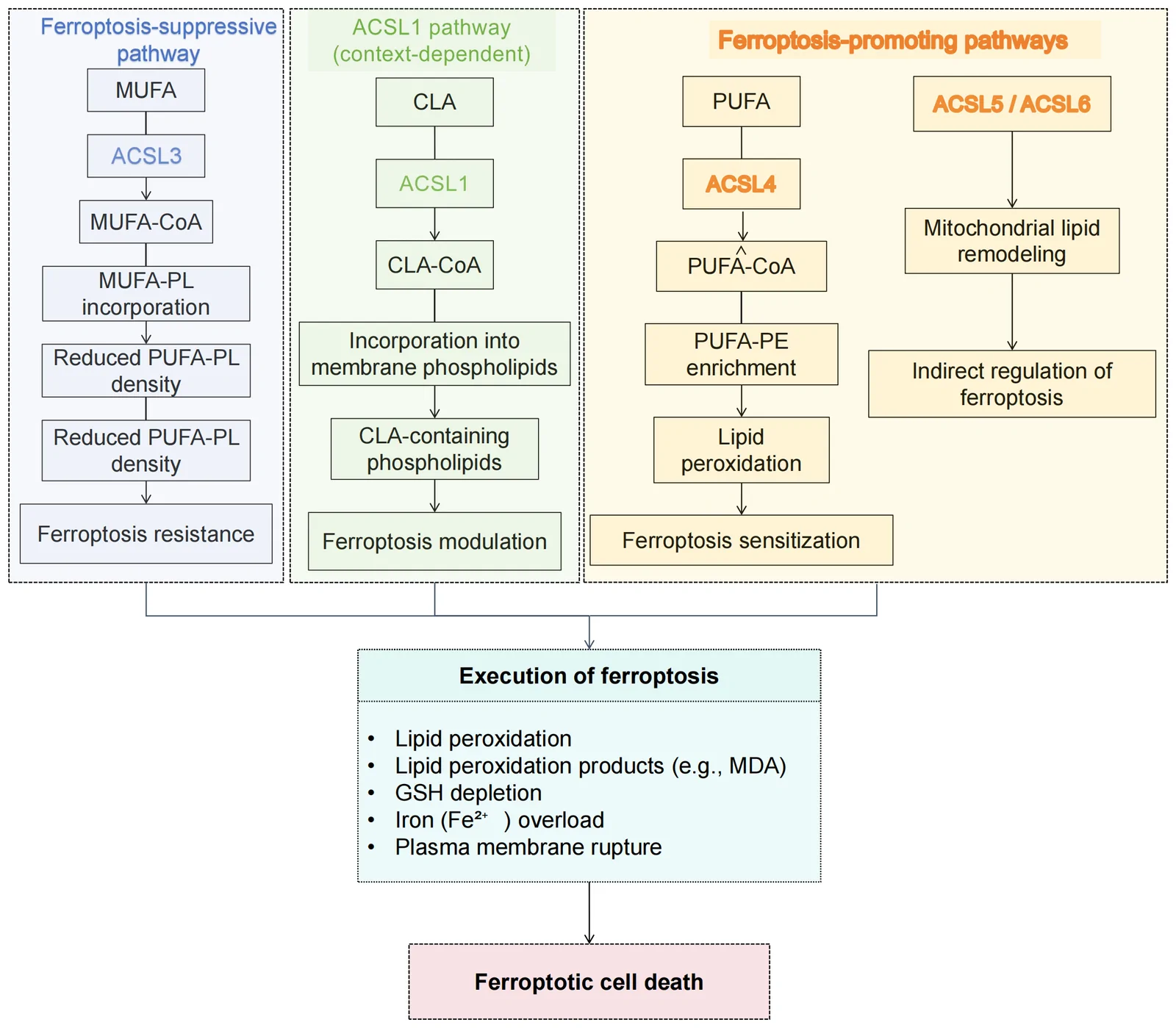
ACSL-mediated lipid remodeling and ferroptosis susceptibility. The diagram summarizes the proposed roles of ACSL isoforms in regulating ferroptosis through lipid remodeling. ACSL3-mediated MUFA incorporation into membrane phospholipids reduces PUFA-containing phospholipid density and is associated with ferroptosis resistance. In contrast, ACSL4-mediated PUFA incorporation into PUFA-PE-enriched phospholipids promotes lipid peroxidation and ferroptosis sensitization. ACSL1 may modulate ferroptosis in a context-dependent manner through CLA-containing phospholipids, whereas ACSL5 and ACSL6 may indirectly affect ferroptosis through mitochondria-associated lipid remodeling. These lipid remodeling processes collectively influence lipid peroxidation susceptibility and the ferroptosis commitment stage, which is characterized by lipid peroxidation, MDA accumulation, GSH depletion, Fe²^+^ overload, and plasma membrane rupture. ACSL, long-chain acyl-CoA synthetase; MUFA, monounsaturated fatty acid; PUFA, polyunsaturated fatty acid; PUFA-PE, polyunsaturated fatty acid-containing phosphatidylethanolamine; PUFA-PL, polyunsaturated fatty acid-containing phospholipid; CLA, conjugated linoleic acid; MDA, malondialdehyde; GSH, glutathione; Fe²^+^, ferrous iron.

### 1.1 ACSL1

ACSL1 is widely recognized as the dominant regulator of the cellular fatty acid oxidation pathway. Its expression is highly concentrated in metabolically active tissues such as liver, heart (20), skeletal muscle (21) and adipose tissue (22). This tissue-specific distribution suggests ACSL1 plays a central role in maintaining systemic energy homeostasis throughout the body. At the molecular level, ACSL1 exhibits broad substrate specificity but efficiently utilizes unsaturated long-chain fatty acids, particularly oleate (C18:1) and linoleate (C18:2) (23), with its enzymatic activity primarily localized to the outer mitochondrial membrane and endoplasmic reticulum. The subcellular localization of ACSL1 decisively influences its function: when located in the outer mitochondrial membrane, it promotes FAO to supply energy; when localized in the endoplasmic reticulum, it drives triglyceride synthesis and lipid storage (24).

The function of ACSL1 extends far beyond lipid metabolism, extensively participating in various pathological processes. In cell death regulation, ACSL1 mediates the incorporation of conjugated linoleic acid into neutral lipids, thereby inducing ferroptosis (25). ACSL1 expression is elevated in EC, and functional studies further demonstrate that ACSL1 overexpression enhances fatty acid β-oxidation and ATP production through the AMPK/CPT1C/ATP axis, thereby promoting EC cell proliferation and migration (8, 26). In CC, ACSL1 methylation levels significantly increase with cervical lesion progression (26). However, its functional consequences for lipid metabolism and tumor behavior remain to be established. These findings indicate that the reproductive relevance of ACSL1 is context dependent rather than uniform. In EC, direct functional evidence supports an ACSL1-driven increase in FAO and ATP production that promotes malignant proliferation and migration, whereas its role in CC is currently supported mainly by epigenetic association. More broadly, ACSL1-mediated partitioning of activated fatty acids between oxidative and lipid-synthetic pathways provides a mechanistic basis for investigating how altered lipid flux contributes to reproductive pathology; however, disease-specific functions require validation in individual reproductive tissues and cell types.

### 1.2 ACSL3

ACSL3 is another long-chain acyl-CoA synthetase with established roles in phosphatidylcholine synthesis, lipid-droplet formation, and monounsaturated-fatty-acid activation (27–31). However, disease-specific evidence across female reproductive diseases (FRDs) is uneven. In cervical cancer (CC), the demonstrated HPV/IGF2BP3/SCD pathway increases oleic-acid production, but ACSL3 itself was not measured (32). Therefore, positioning ACSL3 as the enzyme that channels SCD-derived oleate into membrane lipids is mechanistically plausible but not yet directly established in CC. In ovarian cancer (OC), transcriptomic and prognostic studies report context-dependent associations (33, 34), whereas detailed anti-ferroptotic and lipid-droplet mechanisms have largely been derived from non-ovarian experimental systems (35–37). Mechanistically, ACSL3-dependent activation of monounsaturated fatty acids (MUFAs), particularly oleate, promotes their incorporation into membrane phospholipids and reduces the relative abundance of oxidation-prone PUFA-containing phospholipids, thereby limiting lipid peroxidation and promoting ferroptosis resistance (36). Importantly, this anti-ferroptotic mechanism was established in non-reproductive experimental systems and has not yet been directly validated in FRDs. Accordingly, ACSL3 should currently be viewed as a candidate metabolic node whose disease-specific functions require direct validation. In contrast to its established role in lipid remodeling and ferroptosis resistance, direct disease-specific evidence linking ACSL3 to inflammatory signaling in FRDs remains limited. Thus, whether ACSL3-mediated lipid remodeling directly modulates inflammatory pathways in reproductive tissues remains an important question for future investigation.

### 1.3 ACSL4

ACSL4 is one of the most functionally and substrate-specific isoforms within ACSLs, exhibiting high affinity for PUFAs, particularly AA and eicosapentaenoic acid (EPA) (38). ACSL4 has been reported at the endoplasmic reticulum, including mitochondria-associated ER membranes (MAMs), as well as peroxisomal and other membrane-associated sites. ACSL4 extensively participates in fatty acid activation and downstream metabolic processes, including phospholipid and triglyceride synthesis, cholesterol esterification, and the generation of AA derivatives such as prostaglandins and leukotrienes (39).

Importantly, ACSL4 promotes ferroptosis primarily by promoting the incorporation of PUFAs into membrane phospholipids, thereby increasing the availability of oxidizable substrates for lipid peroxidation (17, 40). Mechanistically, ACSL4 activates PUFAs, particularly AA and AdA, to generate PUFA-CoA derivatives, which are subsequently incorporated into membrane phospholipids, especially phosphatidylethanolamine (PE), through phospholipid remodeling enzymes such as LPCAT3. The resulting PUFA-containing phospholipids are highly susceptible to iron-dependent oxidation, leading to phospholipid hydroperoxide accumulation and enhanced lipid peroxidation, thereby sensitizing cells to ferroptosis (17, 40). Thus, ACSL4 promotes ferroptosis mainly by remodeling membrane phospholipids toward a PUFA-enriched and oxidation-prone state.

In terms of tissue distribution, ACSL4 is highly expressed in the brain, testes, ovaries, and adrenal glands (41). Its enrichment in steroidogenic tissues is consistent with functional evidence linking ACSL4-dependent AA metabolism to hormone-stimulated steroidogenesis (5). In addition, studies in breast cancer models have demonstrated that ACSL4 activity is associated with enhanced proliferation, migration, invasion, metastatic potential, and therapeutic resistance, supporting a context-dependent role for ACSL4-mediated lipid remodeling in aggressive tumor phenotypes (12, 42). Within ovarian tissue, ACSL4 enhances cell membrane fluidity and invasive capacity by catalyzing PUFA synthesis, acting as a key factor promoting hematogenous metastasis in OC (43).

Beyond its role in ferroptosis, ACSL4 also links PUFA remodeling to inflammatory lipid signaling. By preferentially activating AA and facilitating its incorporation into membrane phospholipids, ACSL4 regulates the AA-containing phospholipid pool that serves as a source of substrates for bioactive eicosanoid synthesis through cyclooxygenase (COX) and lipoxygenase (LOX) pathways (18). Consistent with this mechanism, functional studies have shown that ACSL4 deficiency alters AA incorporation into phospholipids and the production of AA-derived inflammatory lipid mediators, including prostaglandins and leukotrienes (44). However, direct disease-specific evidence establishing these ACSL4-dependent inflammatory mechanisms in FRDs remains limited, and their relevance to individual reproductive diseases requires further validation.

In reproductive pathologies, ACSL4 has been implicated in several disease-specific contexts. In endometriosis (EM), ACSL4 influences endometrial receptivity and lesion progression via ferroptosis (45). In ovarian dysfunction associated with hyperandrogenism and endoplasmic reticulum stress, altered ACSL4-related lipid peroxidation and iron homeostasis have been associated with impaired granulosa-cell function (46). ACSL4 is also upregulated in placental tissue from patients with PE and is associated with a ferroptosis-related molecular signature; however, the available clinical tissue data are primarily marker-based and do not by themselves establish trophoblast ferroptotic cell death, warranting further functional and ultrastructural validation (47). Collectively, current evidence supports ACSL4 as an important interface between PUFA metabolism, ferroptosis susceptibility, and inflammatory lipid signaling in female reproductive pathologies, although the strength of disease-specific functional evidence varies across conditions.

### 1.4 ACSL5

ACSL5 is localized to both the outer mitochondrial membrane and the endoplasmic reticulum and participates in the partitioning of activated fatty acids between oxidative and lipid-synthetic pathways (48). Rather than exclusively directing fatty acids toward β-oxidation, ACSL5 exhibits context-dependent metabolic flexibility. Functional studies have shown that ACSL5 can promote the incorporation of fatty acids into triacylglycerols, phospholipids, and cholesterol esters and facilitate lipid-droplet formation, whereas alterations in ACSL5 activity can also shift fatty-acid flux toward oxidation depending on cellular energy and metabolic conditions (49). Thus, the metabolic consequences of ACSL5 activity depend on subcellular localization, tissue context, and substrate availability.

ACSL5 has also been functionally linked to apoptosis in non-reproductive tissues. In intestinal epithelial models, ACSL5 promotes apoptotic susceptibility through ceramide-dependent reduction of the anti-apoptotic protein c-FLIP and increased expression of the death receptor TRAIL-R1 (50). Other studies indicate that the apoptotic consequences of ACSL5 are context dependent and can interact with death-receptor signaling and TP53 status (51). However, comparable ACSL5-dependent apoptotic mechanisms have not yet been directly established in female reproductive tissues.

Overall, direct functional evidence for ACSL5 in FRDs remains limited. Current data do not support assigning ACSL5 a direct ferroptosis-regulatory role comparable to ACSL4 in reproductive tissues. Therefore, its potential involvement in reproductive pathology should currently be regarded as an area requiring disease- and cell-specific functional validation rather than an established mechanism.

### 1.5 ACSL6

ACSL6 is a member of ACSLs with a prominent role in PUFA metabolism and is particularly important for maintaining DHA-containing lipid pools. It is highly expressed in the central nervous system and hematopoietic system (52), and is also detectable in several other tissues (53). ACSL6 is a membrane-associated enzyme that has been localized to the endoplasmic reticulum membrane and has also been reported at the plasma membrane (10). Through the activation of long-chain fatty acids, particularly DHA, ACSL6 contributes to membrane phospholipid remodeling and DHA retention (52).

Recent functional evidence further indicates that ACSL6 can modulate cellular susceptibility to DHA-induced lipid peroxidation and ferroptosis. In colorectal and breast cancer models, ACSL6 overexpression enhanced DHA-induced lipid peroxidation and GPX4 reduction, whereas ACSL6 knockdown attenuated these effects. Importantly, ferrostatin-1 reversed ACSL6-enhanced DHA cytotoxicity, providing functional evidence for the involvement of ACSL6 in DHA-induced ferroptosis (54). The same study showed that ACSL6 suppressed AKT/ERK signaling, reduced cellular ATP levels, and activated AMPK, linking ACSL6-dependent lipid metabolism to cellular bioenergetic and stress responses (54). However, these mechanisms were demonstrated in colorectal and breast cancer models and have not yet been directly established in female reproductive diseases.

At the genetic level, ACSL6 variants and susceptibility haplotypes have been associated with premature ovarian failure (POF) (55). However, this evidence is associative and does not establish a functional link between ACSL6-mediated DHA metabolism and ovarian dysfunction.

Overall, direct mechanistic evidence for ACSL6 in female reproductive pathology remains sparse. Future studies should determine whether ACSL6-dependent DHA remodeling and ferroptosis regulation operate in specific reproductive cell types and disease contexts.

## 2 ACSLs and cervical cancer

CC remains a major gynecological malignancy worldwide, with persistent high-risk human papillomavirus (HPV) infection representing its principal etiological factor (56, 57). Beyond virus-driven oncogenic signaling, metabolic reprogramming has emerged as an important feature of CC progression, including alterations in fatty-acid synthesis, utilization, and membrane-lipid composition. Ferroptosis-related lipid peroxidation and redox regulation have also been increasingly implicated in CC biology and therapeutic response. Within this metabolic context, individual ACSL isoforms show distinct levels of disease-specific evidence: ACSL4 has direct functional support for regulating ferroptosis and treatment sensitivity in CC, ACSL3 is implicated mainly through an inferred oleate-remodeling mechanism, whereas ACSL5 currently has disease-specific transcriptomic association but lacks direct functional validation in cervical tumors. The following sections therefore distinguish established CC-specific mechanisms from associative and hypothesis-generating evidence for ACSL3, ACSL4, and ACSL5(**Figure 2**; **Table 2**), whereas direct disease-specific functional evidence for ACSL1 and ACSL6 remains insufficient; accordingly, the latter isoforms are not discussed as separate mechanistic subsections.

**Figure 2:**
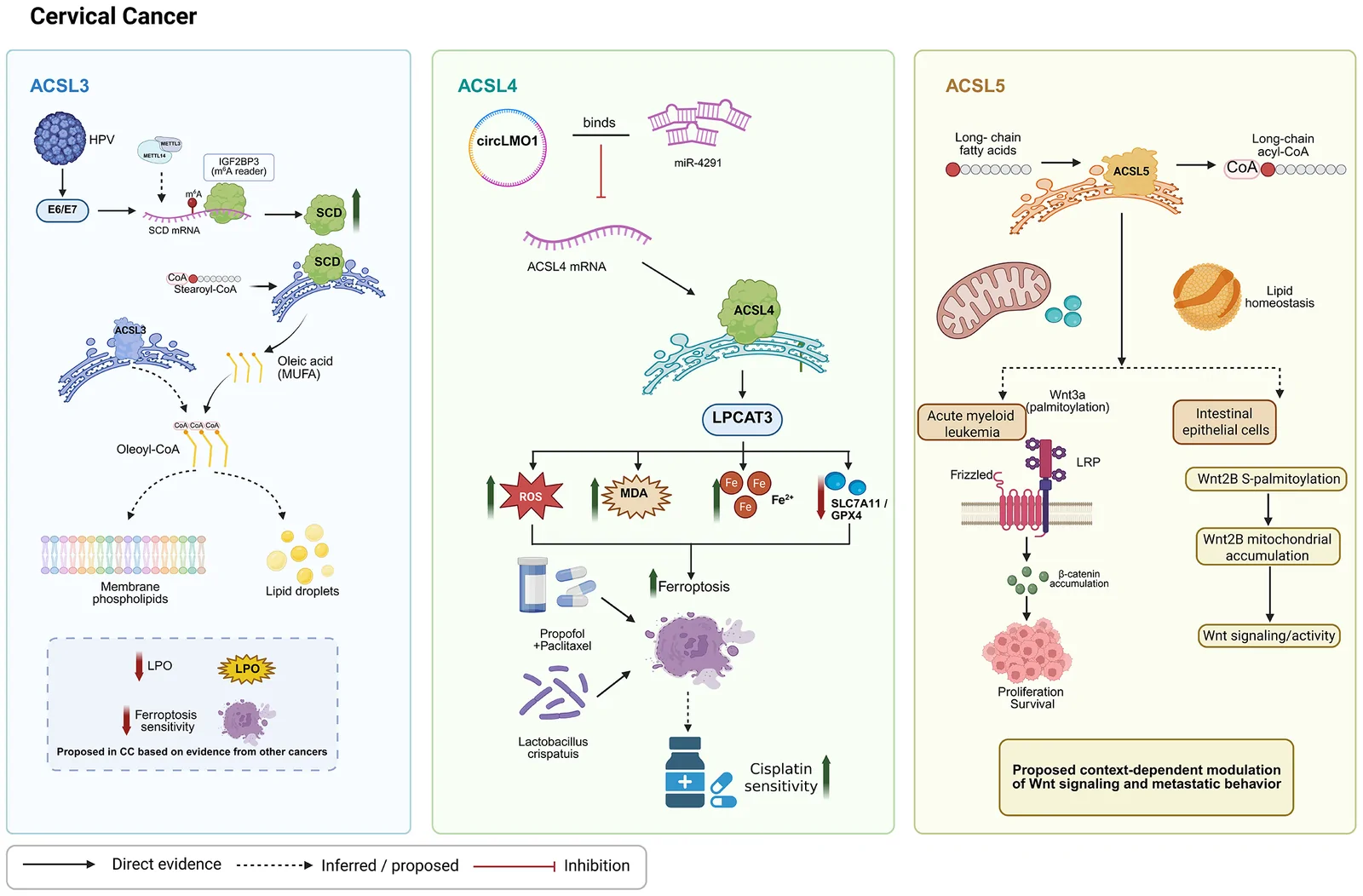
Isoform-specific roles of ACSL3, ACSL4, and ACSL5 in cervical cancer. A proposed model links HPV-driven, SCD-dependent oleic-acid production to ACSL3-mediated membrane phospholipid synthesis, lipid-droplet accumulation, and reduced ferroptosis sensitivity; the HPV/SCD and lipidomic observations are CC-specific, whereas the ACSL3 step is inferred. ACSL4 promotes lipid peroxidation and ferroptosis through the circLMO1/miR-4291–ACSL4–LPCAT3 axis, thereby enhancing cisplatin sensitivity. As for ACSL5, its proposed involvement in Wnt/β-catenin signaling is mainly extrapolated from AML or metabolic-disease studies. Solid arrows indicate disease-specific direct evidence; dashed arrows indicate associative, inferred, or proposed relationships; red lines indicate inhibition. ACSL, long-chain acyl-CoA synthetase; AML, acute myeloid leukemia; CC, cervical cancer; CoA, coenzyme A; Fe²^+^, ferrous iron; GSH, glutathione; HPV, human papillomavirus; LPO, lipid peroxidation; LPCAT3, lysophosphatidylcholine acyltransferase 3; LRP, low-density lipoprotein receptor-related protein; m6A, N6-methyladenosine; MDA, malondialdehyde; MUFA, monounsaturated fatty acid; ROS, reactive oxygen species; SCD, stearoyl-CoA desaturase.

**Table 2:** The roles of ACSLs in different pathways and female reproductive diseases.

Pathway type	ACSL	Associated diseases	Core mechanisms	Diseases with direct experimental evidence	Diseases potentially involved (hypothesis-based)
Ferroptosis	ACSL4	CC, OC, PCOS, EM, EC, PE	Increased PUFA incorporation into membrane phospholipids → lipid peroxidation accumulation → GPX4 suppression → ferroptosis	CC, OC, PCOS, EM, EC, PE	
Fatty acid oxidation (FAO)/energy metabolism	ACSL1, ACSL5, ACSL6	CC, OC, PCOS, EM, EC, PE	Enhanced fatty acid activation and mitochondrial FAO → ATP production ↑ → metabolic reprogramming, proliferation and migration	OC, EC	CC, PCOS, EM, PE
Lipid homeostasis/MUFA-related pathways	ACSL3, ACSL6	CC, OC, PCOS, EM, EC, PE	Increased oleic acid and MUFA metabolism → membrane stabilization, lipid droplet formation and resistance to ferroptosis		CC, OC, PCOS, EM, EC, PE
Epigenetic regulation/m6A modification	ACSL4, ACSL3, ACSL5	CC, EM, PE	METTL3–IGF2BP3–YBX1–mediated m6A modification regulating ACSL transcription and mRNA stability	PE	CC, EM
Inflammation and signaling cascades	ACSL1, ACSL5	CC, OC, PCOS, EM, EC, PE	Activation of inflammatory and metabolic signaling pathways, including IL-6/JAK/STAT3, p38 MAPK and PPARδ, leading to disease progression		CC, OC, PCOS, EM, EC, PE

This table summarizes ACSL-associated pathways in female reproductive diseases. “Direct evidence” denotes disease-specific patient, tissue, cell, organoid, or animal data that measure or manipulate the named ACSL isoform. Disease-level pathway evidence that does not test the named isoform, bioinformatic association, cross-disease evidence, and cross-species evidence are classified as indirect or hypothesis-generating. Evidence Hierarchy of ACSL Isoforms Across FRDs--ACSL1: Direct evidence: OC, EC. Indirect evidence: CC. Research gaps: PCOS, EM, PE. ACSL3: Direct evidence: None. Indirect evidence: CC, OC, PCOS, EM, PE. Research gap: EC. ACSL4: Direct evidence: CC, OC, PCOS, EM, EC, PE. Indirect evidence: None. Research gaps: None among the six major FRDs reviewed. ACSL5: Direct functional evidence: None. Indirect evidence: CC, OC, EM, PE. Research gaps: PCOS, EC. EM has direct disease-specific expression evidence, but no direct functional validation. ACSL6: Direct evidence: None among the six major FRDs reviewed. Indirect evidence: POF, mainly based on genetic association. Research gaps: CC, OC, PCOS, EM, EC, PE.

### 2.1 ACSL3: the role in cervical cancer

Direct evidence that ACSL3 is overexpressed, prognostic, or functionally required in CC remains limited. Disease-specific studies instead demonstrate that IGF2BP3-dependent stearoyl-CoA desaturase (SCD) expression increases oleic-acid production, that the cervicovaginal lipid environment is altered, and that HPV remodels host fatty-acid metabolism (32, 58, 59). Because ACSL3 can activate MUFAs such as oleate and promote their incorporation into cellular lipids in other experimental systems, an HPV/IGF2BP3/SCD–oleate–ACSL3 pathway can be proposed as a hypothesis for CC lipid remodeling. However, ACSL3 itself was not examined in the CC studies supporting the upstream pathway, and this proposed mechanism therefore remains unvalidated. Evidence for ACSL3-mediated lipid storage and ferroptosis resistance is derived predominantly from non-cervical cancer models (28, 35). Thus, ACSL3 should currently be considered a candidate metabolic node in CC rather than an established disease driver (**Figure 2**; **Table 2**).

### 2.2 ACSL4: the role in cervical cancer

A recent study showed that combined propofol and paclitaxel treatment induced ferroptosis-associated changes in C-33A and HeLa cervical cancer cells, accompanied by increased ACSL4 expression (60). A metabolomic and proteomic study further showed that *Lactobacillus crispatus*, a major commensal species of the healthy cervicovaginal microbiota, inhibited SiHa cervical cancer cells in association with ferroptosis-related metabolic changes, including altered ACSL4 and LPCAT3 expression, activation of the TCA cycle, and increased ROS and lipid-peroxide levels (61).

In CC tissues, cell models, and xenografts, circLMO1 has been identified as an upstream regulator of ACSL4-dependent ferroptosis. Mechanistically, circLMO1 functions as a competing endogenous RNA that sponges miR-4291, thereby relieving miR-4291-mediated suppression of ACSL4. Increased ACSL4 expression promotes ferroptosis and suppresses CC cell growth and metastatic behavior, whereas ACSL4 knockdown reverses these effects (62). Clinically, the relevance of ACSL4 extends to chemosensitivity. In cervical squamous cell carcinoma, ACSL4 upregulation correlates closely with lipid ROS, Fe²^+^, and MDA accumulation, accompanied by downregulation of both solute carrier family 7 member A11 (SLC7A11) and GPX4, thereby enhancing cellular sensitivity to cisplatin (63). Collectively, these findings reveal ACSL4’s important role in ferroptosis susceptibility and treatment response. This suggests that targeting ACSL4 or its regulatory pathways may represent novel strategies to enhance therapeutic sensitivity in CC treatment.

### 2.3 ACSL5: the role in cervical cancer

Disease-specific evidence for ACSL5 in CC remains limited and is currently association based. A recent RNA-sequencing study identified ACSL5 as one of four genes included in a predictive model for lymph-node metastasis in CC (64). This finding establishes a disease-specific association between ACSL5 and metastatic-risk prediction but does not demonstrate that ACSL5 functionally drives cervical cancer metastasis. Mechanistic studies in non-cervical systems nevertheless indicate that ACSL5 can modulate Wnt signaling in a context-dependent manner. In acute myeloid leukemia, ACSL5 promotes Wnt/β-catenin signaling through Wnt3a palmitoylation, whereas ACSL5 knockdown suppresses Wnt/β-catenin activity and leukemic cell growth (65). In contrast, an intestinal epithelial study showed that ACSL5-dependent S-palmitoylation of Wnt2B promoted its mitochondrial accumulation and was associated with reduced Wnt activity (66). These apparently divergent findings indicate that ACSL5-dependent regulation of Wnt signaling is strongly dependent on cellular context. Importantly, neither mechanism has been demonstrated in cervical cancer. Therefore, whether ACSL5 modulates Wnt signaling or metastatic behavior in CC should be regarded as a testable hypothesis rather than an established disease mechanism. Direct manipulation of ACSL5 in cervical cancer models will be required to address this possibility (**Figure 2**; **Table 2**).

## 3 ACSLs and ovarian cancer

Ovarian cancer (OC) is a heterogeneous group of gynecological malignancies with distinct histological and molecular characteristics. Epithelial ovarian cancer (EOC) represents the predominant category, among which high-grade serous ovarian cancer (HGSOC) is the most common subtype (67, 68). Increasing evidence indicates that lipid metabolic reprogramming and ferroptosis-related regulation contribute to OC progression, metastatic adaptation, and therapeutic response. However, the strength of evidence differs substantially among ACSL isoforms. Current disease-specific functional evidence is strongest for ACSL1 and ACSL4, whereas the evidence for ACSL3 and ACSL5 remains predominantly transcriptomic or prognostic. Direct disease-specific evidence for ACSL6 in ovarian cancer remains insufficient, and ACSL6 is therefore not discussed as a separate mechanistic subsection. These isoform-specific findings and their corresponding evidence levels are summarized in **Table 2**.

### 3.1 ACSL1: the role in ovarian cancer

Metabolic reprogramming contributes to OC progression (69), and functional studies have identified ACSL1 as an important regulator of fatty-acid metabolism during metastatic adaptation. Proteomic analysis comparing highly metastatic (HM) and non-metastatic (NM) OC cells showed increased fatty-acid metabolic activity and enrichment of phospholipids containing relatively shorter long-chain fatty acids, including myristic acid (C14:0), in HM cells. ACSL1 overexpression shifted the lipid profile of NM cells toward that of HM cells and enhanced invasive behavior. Mechanistically, ACSL1-dependent myristoyl-CoA production promoted protein N-myristoylation, activating AMPK/Src signaling and enhancing fatty-acid oxidation (FAO). Consistent with these functional findings, ACSL1 expression was higher in omental metastases than in primary tumors and was associated with poorer clinical outcome (70).

Histological context should nevertheless be considered when interpreting ACSL1-associated metastatic adaptation. In a separate xenograft study using the ES-2 ovarian clear cell carcinoma (OCCC) cell line, ACSL1, ferritin light chain (FTL), and lactate dehydrogenase A (LDHA) were specifically upregulated in liver metastases, suggesting metabolic adaptation of OCCC cells to the hepatic microenvironment (71). Because this evidence derives specifically from an OCCC model, it should not be generalized to HGSOC or other epithelial OC subtypes. Thus, current evidence supports ACSL1-dependent metabolic remodeling and ferroptosis resistance in OC (72), while the extent to which these mechanisms vary across histological subtypes requires further investigation (**Figure 3**).

**Figure 3:**
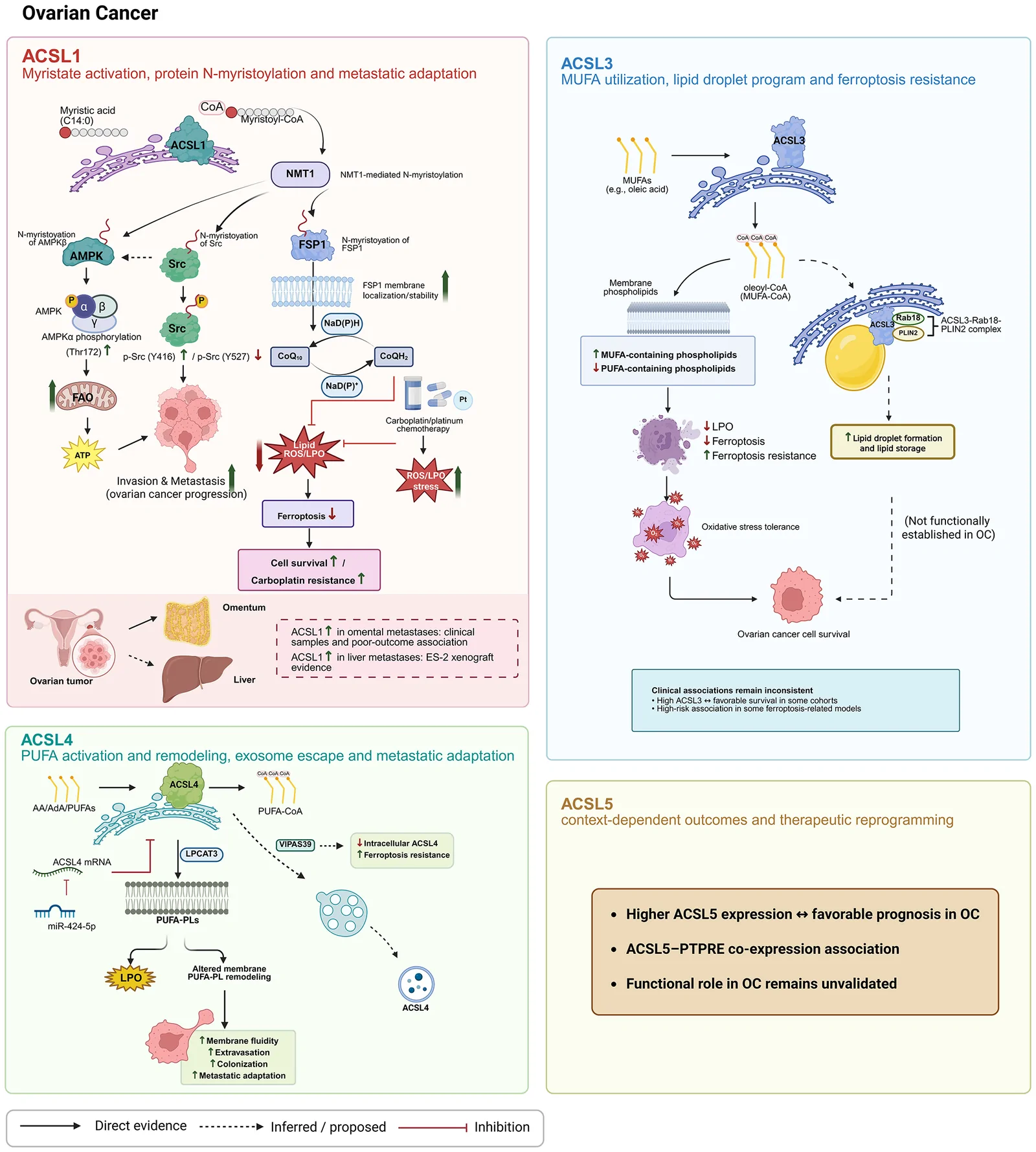
Isoform-specific roles of long-chain acyl-CoA synthetases in ovarian cancer. ACSL1 promotes myristoyl-CoA production, protein N-myristoylation, FAO, metastatic progression, and FSP1-dependent ferroptosis/platinum resistance in OC models. ACSL3 has disease-specific prognostic associations, but its MUFA, membrane-phospholipid, lipid-droplet, and ferroptosis mechanisms are largely inferred from non-OC systems. ACSL4 regulates PUFA-phospholipid remodeling, VIPAS39-dependent exosomal escape from ferroptosis, and metastatic adaptation. ACSL5 evidence in OC is primarily prognostic/co-expression based; mechanistic claims are not yet established. Solid arrows indicate disease-specific direct evidence; dashed arrows indicate associative, inferred, or proposed relationships; red lines indicate inhibition. AA, arachidonic acid; ACSL, long-chain acyl-CoA synthetase; AdA, adrenic acid; AKT, protein kinase B; AMPK, AMP-activated protein kinase; ATP, adenosine triphosphate; CoA, coenzyme A; CoQ_1__0_, coenzyme Q_1__0_; CoQH_2_, reduced coenzyme Q_1__0_; ER, endoplasmic reticulum; FAO, fatty acid oxidation; FASN, fatty acid synthase; FSP1, ferroptosis suppressor protein 1; LPO, lipid peroxidation; LPCAT3, lysophosphatidylcholine acyltransferase 3; MUFA, monounsaturated fatty acid; NAD(P)H, reduced nicotinamide adenine dinucleotide (phosphate); NMT1, N-myristoyltransferase 1; OC, ovarian cancer; PI3K, phosphatidylinositol 3-kinase; PL, phospholipid; PLIN2, perilipin 2; PUFA, polyunsaturated fatty acid; Rab18, Ras-related protein Rab-18; ROS, reactive oxygen species; VIPAS39, VPS33B-interacting protein, apical–basolateral polarity regulator, spe-39 homolog.

### 3.2 ACSL3: the role in ovarian cancer

Evidence linking ACSL3 to OC remains predominantly transcriptomic and prognostic rather than functional. A systematic pan-cancer analysis reported that higher ACSL3 expression was associated with better prognosis in ovarian cancer, whereas a separate TCGA-based ferroptosis-related prognostic model included ACSL3 among a multigene signature associated with patient outcome (32, 33). However, these studies differ substantially in cohort composition, analytical design, and the manner in which ACSL3 was evaluated, and neither provides direct functional evidence establishing ACSL3 as a tumor-promoting or tumor-suppressive factor in a defined OC histological subtype. The available data should therefore be interpreted as prognostic associations rather than evidence of a specific causal role. Mechanistic studies outside OC have shown that ACSL3-dependent activation and incorporation of monounsaturated fatty acids can reduce the relative abundance of oxidation-prone PUFA-containing membrane phospholipids and thereby suppress ferroptosis, while ACSL3 can also cooperate with Rab18 and PLIN2 in lipid-droplet dynamics (36, 37). These mechanisms provide a biochemical basis for future investigation but have not been directly demonstrated in OC. Disease- and subtype-specific perturbation studies will therefore be required to determine whether ACSL3-mediated MUFA remodeling or lipid-droplet regulation contributes to ovarian tumor biology (**Figure 3**).

### 3.3 ACSL4: the role in ovarian cancer

ACSL4 has comparatively strong disease-specific functional evidence in OC, particularly in the regulation of PUFA-phospholipid remodeling and ferroptosis susceptibility. In epithelial ovarian cancer (EOC) models, sensitivity to IFN-γ/arachidonic acid (AA)-induced ferroptosis varies among cell lines. Ferroptosis-resistant EOC cells can reduce intracellular ACSL4 by exporting the protein through exosomes. VIPAS39 facilitates ACSL4 sorting into late endosomes and subsequent exosomal release, whereas VIPAS39 depletion retains intracellular ACSL4, increases ferroptosis sensitivity, and suppresses tumor growth in xenograft models (73). These findings identify regulated ACSL4 export as a disease-specific mechanism of ferroptosis evasion in EOC.

Additional OC studies support ACSL4 as a functional determinant of ferroptosis susceptibility. Portulaca oleracea L. polysaccharide (POL) increased ACSL4 expression and ferroptosis-associated lipid oxidation in OC cells and xenografts, and ACSL4 knockdown reversed the associated lipid ROS, Fe²^+^, MDA, and GSH changes (74). N-(4-morpholinomethylene)ethanesulfonamide (MESA) also induced ferroptotic cell death in ovarian cancer cells, accompanied by ACSL4 upregulation and alterations in the SLC7A11/GPX4 and NRF2-related antioxidant pathways (75). Obacunone similarly increased ACSL4 while decreasing GPX4 and GSH and modulating Akt/p53 signaling in OC models (76). In addition, miR-424-5p directly targets the ACSL4 3′-UTR and reduces erastin- and RSL3-induced ferroptosis, whereas restoration of ACSL4 reverses this effect (77).

ACSL4-dependent PUFA remodeling is also relevant to metastatic adaptation. A recent study demonstrated that metastatic OC-derived cells contain higher PUFA-lipid levels and exhibit greater ferroptosis susceptibility than primary-tumor-derived cells. Functional CRISPR screening further identified ACSL4 as a driver of hematogenous metastatic extravasation by increasing PUFA-lipid abundance, membrane fluidity, and cellular invasiveness. This PUFA-enriched state simultaneously creates increased dependence on enzymes required for unsaturated-fatty-acid β-oxidation, including ECH1, and combined ACSL4/ECH1 inhibition markedly suppresses metastasis (43). Thus, rather than implying separate or contradictory functions, current evidence supports a context-dependent coupling in which ACSL4-driven PUFA remodeling enhances membrane plasticity and metastatic adaptation while also increasing the abundance of oxidation-prone lipids and ferroptosis susceptibility. This mechanistic relationship helps reconcile the metastatic and ferroptosis-related observations without assuming that ACSL4 uniformly promotes or suppresses OC progression (**Figure 3**).

### 3.4 ACSL5: the role in ovarian cancer

Available evidence for ACSL5 in OC remains predominantly bioinformatic. A systematic analysis of ACSL family members reported that higher ACSL5 expression was associated with favorable prognosis in ovarian cancer and identified co-expression patterns including protein tyrosine phosphatase receptor type E (PTPRE) (33). However, these findings establish prognostic and co-expression associations rather than a functional ACSL5–PTPRE pathway. Direct perturbation studies in OC cells, organoids, or *in vivo* models are currently lacking; therefore, ACSL5 should not yet be assigned a tumor-suppressive mechanism or considered a validated therapeutic target in OC. Together with the stronger functional evidence for ACSL1 and ACSL4 and the predominantly associative evidence for ACSL3, this evidence hierarchy is summarized in the corresponding figure and table (**Figure 3**; **Table 2**).

## 4 ACSLs and polycystic ovary syndrome

PCOS is a common endocrine and metabolic disorder predominantly affecting women of reproductive age, with a global prevalence of approximately 6%–20% (78). Its clinical presentation is highly heterogeneous, with key features including hyperandrogenism, ovulatory dysfunction, and polycystic ovarian morphology (79). In addition to menstrual irregularities and infertility, PCOS is frequently associated with insulin resistance, obesity, type 2 diabetes, and increased cardiovascular disease risk (80). Its pathogenesis is multifactorial and involves complex interactions among genetic, epigenetic, endocrine, and metabolic factors. Current management therefore combines lifestyle interventions with pharmacological approaches tailored to metabolic and reproductive manifestations (81). Among individual ACSL isoforms, direct and reproducible disease-specific evidence for ACSL1, ACSL5, and ACSL6 in PCOS remains insufficient; therefore, the following discussion focuses on ACSL3 and ACSL4, for which comparatively more relevant evidence is currently available.

### 4.1 ACSL3: the role in polycystic ovary syndrome

Current PCOS studies cited here do not directly quantify or experimentally perturb ACSL3. Instead, they show that oleate/palmitate exposure, hepatic HNF-4α signaling, dietary fatty-acid composition, and metabolic interventions can modify insulin resistance, hepatic lipid handling, or reproductive phenotypes (82–84). Because ACSL3 preferentially activates oleate, these observations motivate an ACSL3-centered liver–ovary metabolic hypothesis, but they do not validate ACSL3 as the mediator of the reported effects.

Importantly, hepatic lipid dysregulation and local ovarian lipotoxicity should be distinguished mechanistically. Altered hepatic lipid handling may contribute to systemic insulin resistance and endocrine disturbances, whereas excessive fatty-acid exposure within the ovarian microenvironment can directly impair granulosa-cell function and folliculogenesis. However, direct evidence linking ACSL3 to either process in PCOS remains lacking. Accordingly, ACSL3 should currently be regarded as a candidate metabolic node rather than an established regulator of PCOS. Future studies should directly quantify ACSL3 in patient-derived tissues and use selective gain- or loss-of-function approaches in hepatocyte and granulosa-cell models to determine whether ACSL3-mediated MUFA activation independently contributes to hepatic or ovarian lipid dysregulation in PCOS.

### 4.2 ACSL4: the role in polycystic ovary syndrome

ACSL4 currently has the strongest disease-specific evidence among the ACSL isoforms discussed in PCOS, particularly in relation to lipid peroxidation and ferroptosis-associated reproductive injury. In uterine tissue from a PCOS-like pregnancy model, combined dihydrotestosterone (DHT) and insulin exposure altered ACSL4 expression and was accompanied by GPX4/GSH depletion, increased MDA, iron deposition, and ferroptosis-associated mitochondrial morphological changes (7). In contrast, placental alterations were more limited, suggesting tissue-dependent responses to hyperandrogenic and metabolic stress. These findings support an association between ACSL4-related lipid-peroxidation pathways and uterine reproductive dysfunction, although the marker changes alone do not establish ACSL4 as the sole causal mediator of ferroptotic cell death.

More direct evidence has been obtained in granulosa-cell models. Elevated palmitic acid in follicular fluid promotes ER stress and ATF4/TXNIP signaling, accompanied by increased ACSL4 and enhanced ferroptosis-related injury in granulosa-cell and ovarian models (46). Beyond ER stress-mediated regulation, DHEA-induced hyperandrogenic stress increases ACSL4 expression and is accompanied by reduced GPX4 and SLC7A11 expression and increased Fe²^+^ accumulation in KGN cells (85).

A further regulatory mechanism involves iron metabolism and mitophagy. TFRC-mediated iron uptake increases ROS production through NOX1 and activates PINK1-associated mitophagy; importantly, iron-induced ACSL4 upregulation is required for mitophagy activation, GPX4 degradation, lipid peroxidation, and ferroptosis in KGN cells (86). These findings link ACSL4 to iron-dependent oxidative stress and mitochondrial quality-control pathways in PCOS-related granulosa-cell injury without implying direct translocation of ACSL4 into mitochondria.

Interventions targeting ferroptosis-related pathways may counteract these ACSL4-associated alterations. 1,25-Dihydroxyvitamin D3 (1,25D3) alleviates hyperandrogen-induced ferroptosis in KGN cells by attenuating the DHEA-induced increase in ACSL4 expression while restoring GPX4 and SLC7A11 expression (85). The natural compound nuciferine similarly attenuates DHEA-induced ferroptotic injury in ovarian granulosa cells by upregulating SRY-Box Transcription Factor 2 (SOX2) and activating the downstream SLC7A11/GPX4 antioxidant axis. Nuciferine also reverses the DHEA-associated increase in ACSL4 expression; however, the demonstrated SOX2-dependent protective mechanism primarily involves SLC7A11/GPX4 rather than direct inhibition of ACSL4 (87). In addition, direct ACSL4 knockdown reduces lipid peroxidation and ferroptotic injury associated with TFRC/NOX1/PINK1 signaling, further supporting the functional involvement of ACSL4 in this experimental context (86).

In summary, ACSL4 is an important component of ferroptosis-related pathways in uterine and ovarian PCOS models, with its regulation linked to ER stress, hyperandrogenic signaling, and iron metabolism. Current evidence indicates that ACSL4-associated lipid peroxidation can contribute to granulosa-cell dysfunction, whereas interventions such as 1,25D3 and nuciferine can suppress ferroptotic injury through distinct regulatory mechanisms. Nevertheless, these interventions should not be interpreted as direct ACSL4 inhibitors unless such activity is experimentally demonstrated. Further studies are needed to determine the tissue- and disease-context-specific regulation of ACSL4 and to evaluate whether ACSL4 or related ferroptosis-regulatory pathways represent feasible therapeutic targets in PCOS (**Figure 4**; **Table 2**).

**Figure 4:**
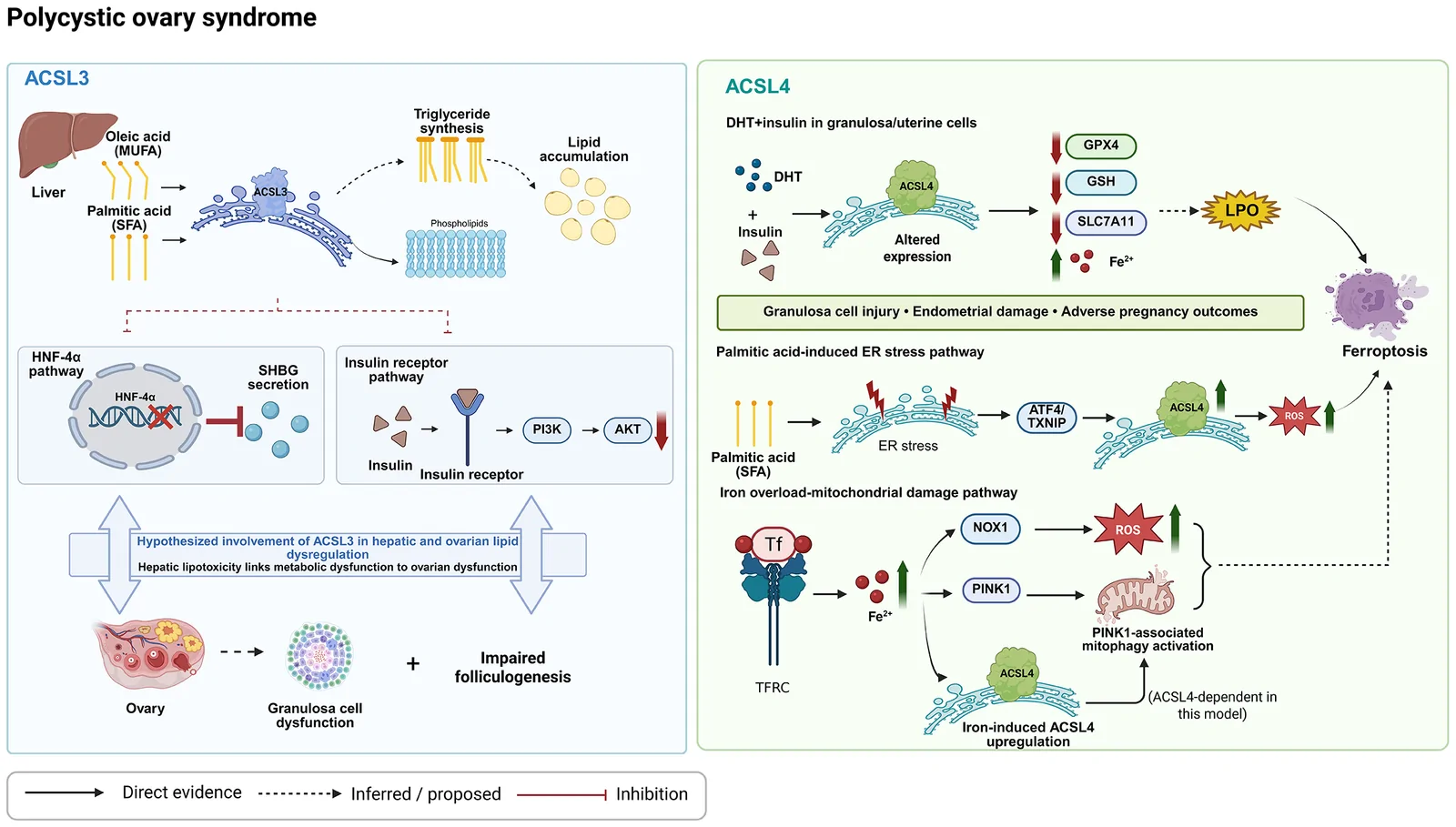
Isoform-specific roles of ACSL3 and ACSL4 in polycystic ovary syndrome. ACSL3 is proposed to connect oleate handling with hepatic lipotoxicity, HNF-4α/insulin signaling, and ovarian dysfunction, but the cited PCOS studies do not directly test ACSL3. ACSL4 promotes ferroptosis through androgen/insulin-associated impairment of the GPX4/GSH/SLC7A11 system, palmitate-induced ATF4/TXNIP signaling, and TFRC/NOX1/PINK1-associated iron and mitochondrial injury. Solid arrows indicate disease-specific direct evidence; dashed arrows indicate associative, inferred, or proposed relationships; red lines indicate inhibition. ACSL3, long-chain acyl-CoA synthetase 3; ACSL4, long-chain acyl-CoA synthetase 4; AKT, protein kinase B; ATF4, activating transcription factor 4; DHT, dihydrotestosterone; ER, endoplasmic reticulum; Fe²^+^, ferrous iron; GPX4, glutathione peroxidase 4; GSH, glutathione; HNF-4α, hepatocyte nuclear factor 4 alpha; LPO, lipid peroxidation; MUFA, monounsaturated fatty acid; NOX1, NADPH oxidase 1; PCOS, polycystic ovary syndrome; PI3K, phosphatidylinositol 3-kinase; PINK1, PTEN-induced kinase 1; ROS, reactive oxygen species; SFA, saturated fatty acid; SHBG, sex hormone-binding globulin; SLC7A11, solute carrier family 7 member 11; Tf, transferrin; TFRC, transferrin receptor; TXNIP, thioredoxin-interacting protein.

## 5 ACSLs and endometriosis

EM is a common chronic gynecological condition affecting approximately 5%–10% of women of reproductive age worldwide. Its hallmark feature is the presence of endometrioid tissue outside the uterine cavity, leading to significant health burdens such as pelvic pain, infertility, and recurrent surgeries (88). The traditional “retrograde menstruation theory” fails to account for its diverse clinical manifestations. Current understanding increasingly attributes it to a multifactorial interplay involving genetic predisposition, environmental factors, immune dysfunction, and estrogen dependence (89). Recent studies indicate that ectopic lesions not only exhibit aromatase activity and progesterone resistance but also involve angiogenesis and chronic inflammation, leading to fibrosis, pain sensitization, and distal neurological symptoms (90). More importantly, it is now recognized as a systemic disorder that further impacts liver and adipose tissue metabolism, induces systemic inflammation, and causes mood disorders and hyperalgesia by altering brain gene expression (88). Current treatments primarily involve hormone suppression and surgical resection, both of which have limitations. The emergence of new-generation oral Gonadotropin-Releasing Hormone (GnRH) antagonists and deepening etiological research hold promise for advancing personalized and precision therapies (91). Direct disease-specific evidence for ACSL1 and ACSL6 in EM is also lacking (**Table 2**).

### 5.1 ACSL3: the role in endometriosis

Direct evidence linking ACSL3 to human endometriosis is currently lacking. Endometriosis studies establish apoptosis resistance and cell-type-specific ferroptosis susceptibility in an iron-rich inflammatory environment (92–94). Findings from vascular endothelial and pancreatic-cancer models suggest that ACSL3-dependent MUFA/lipid-droplet handling or autophagy can support stress resistance (95, 96), but these findings are not disease-specific. Accordingly, whether ACSL3-dependent MUFA and lipid-droplet metabolism alters lipid-peroxidation susceptibility in ectopic endometrial cells remains a testable hypothesis requiring direct lesion- and cell-specific validation.

### 5.2 ACSL4: the role in endometriosis

The expression pattern of ACSL4 in EM remains controversial. Some studies demonstrate its significant upregulation in ectopic endometrial tissue (45), while others fail to observe expression differences (97). This discrepancy may be attributed to variations in sample size, tissue compartment, or patient characteristics. Despite this controversy, available studies collectively suggest an association between ACSL4, lipid peroxidation, and ferroptosis-related processes in EM, although the strength of ferroptosis-specific evidence varies among studies. Elevated ACSL4 expression in the endometrium of patients with endometriosis has been associated with impaired endometrial receptivity, while inhibition of ACSL4 improves receptivity markers, ultrastructure, and endometrial function (45).

Available endometriosis studies suggest that ACSL4-related changes are associated with ferroptosis-related cellular phenotypes. Resveratrol was reported to promote ferroptosis through the miR-21-3p/p53/SLC7A11 pathway, with ACSL4 measured as a ferroptosis-related marker (98). Silencing FZD7 increased ACSL4 expression and lipid ROS levels, whereas ferrostatin-1 rescued several cellular phenotypes (99). In patient-derived endometrial stromal cells, SEMA3F overexpression was associated with reduced ACSL4, Fe²^+^, MDA, IL-6, and TNF-α levels (100). However, these findings support an association between ACSL4 and ferroptosis-related responses rather than establishing ACSL4-specific causal regulation.

In summary, existing studies associate ACSL4 with impaired endometrial receptivity and ferroptosis-related cellular phenotypes in endometriosis. The direction and cell-specific role of ACSL4 remain to be clarified, and further ACSL4-specific validation is required before ACSL4 can be considered a therapeutic target.

### 5.3 ACSL5: the role in endometriosis

Compared with ACSL4, the role of ACSL5 in endometriosis remains less well characterized. Nevertheless, recent transcriptomic evidence supports a disease-specific association between ACSL5 and metabolic alterations in endometriotic lesions. Single-cell RNA sequencing of paired ectopic and eutopic endometrium from women with endometriosis identified cell-type-specific metabolic reprogramming and showed that ACSL5, which participates in the conversion of fatty acids to acyl-CoA, was upregulated in endothelial cells of ectopic lesions compared with paired eutopic endometrium (101). This finding provides direct disease-specific expression evidence for ACSL5, although it does not establish a causal role in lesion survival or ferroptosis.

Consistent with a potential metabolic association, an independent integrative bioinformatics analysis identified ACSL5 among the core lipid-metabolism-related genes associated with endometriosis (102). However, this evidence is transcriptomic and associative rather than functional. In addition, iron-overloaded follicular fluid from patients with endometriosis has been shown to induce NCOA4-dependent ferritinophagy and ferroptosis in granulosa cells, contributing to impaired oocyte maturation. An EM study found that iron-overloaded follicular fluid triggered granulosa-cell ferroptosis through ferritinophagy and impaired oocyte maturation (103); importantly, ACSL5 was not examined in that study. Therefore, current evidence supports altered ACSL5 expression in endometriosis-associated tissues but does not yet establish ACSL5 as a direct regulator of ferroptosis or endometriosis-associated infertility. Functional studies involving ACSL5-specific manipulation in endometriotic lesions and relevant cell populations are required to define its mechanistic role (**Table 2**; **Figure 5**).

**Figure 5:**
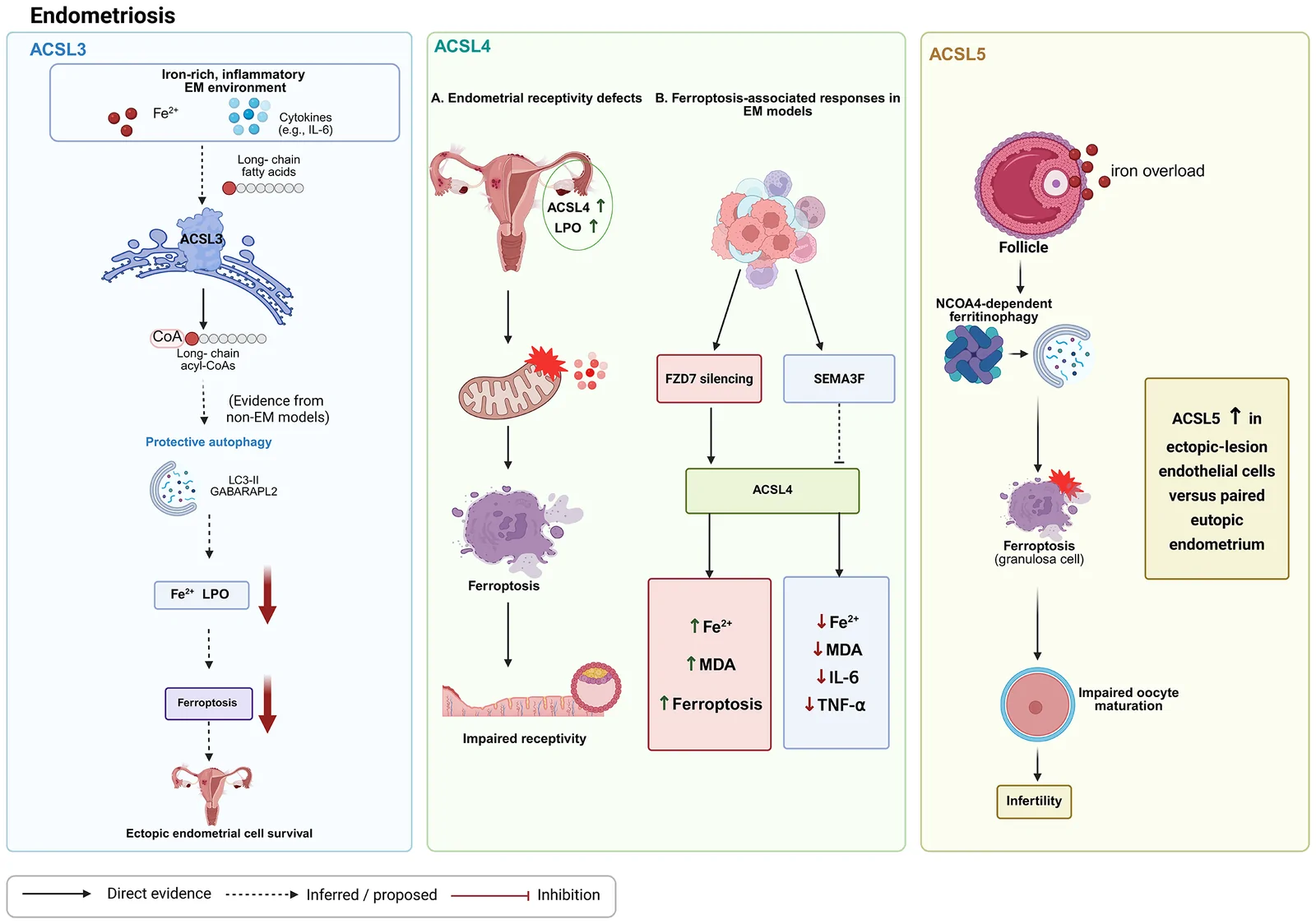
Isoform-specific roles of ACSL3, ACSL4, and ACSL5 in endometriosis. ACSL3-related stress resistance is inferred from non-endometriosis models. ACSL4 is associated with impaired endometrial receptivity and ferroptosis-related responses, whereas ACSL5 has expression and transcriptomic evidence without functional validation. Solid arrows indicate experimental support within specified models; dashed arrows indicate associative, inferred, or proposed relationships; red lines indicate inhibition. ACSL3, long-chain acyl-CoA synthetase 3; ACSL4, long-chain acyl-CoA synthetase 4; ACSL5, long-chain acyl-CoA synthetase 5; AURKB, aurora kinase B; AKT, protein kinase B; EM, endometriosis; Fe²^+^, ferrous iron; FZD7, frizzled class receptor 7; GSH, glutathione; LPO, lipid peroxidation; mTOR, mechanistic target of rapamycin; PI3K, phosphatidylinositol 3-kinase; SEMA3F, semaphorin 3F.

## 6 ACSLs and endometrial cancer

Endometrial cancer (EC) is a common gynecological malignancy whose incidence has increased globally over recent decades (104). Obesity is one of the major modifiable risk factors for EC, with excess adiposity contributing to carcinogenesis through hormonal, metabolic, and inflammatory mechanisms (105). Lipid-metabolic reprogramming is increasingly recognized as an important feature of EC. EC tissues exhibit alterations in fatty-acid composition and increased expression of enzymes involved in *de novo* fatty-acid synthesis and desaturation, including ACACA, FASN, and SCD1 (101, 106). In EC cells, inhibition of sterol regulatory element-binding protein (SREBP)-dependent lipogenesis reduces free-fatty-acid and cholesterol levels and suppresses cell viability, colony formation, and migration. These observations provide a disease-specific metabolic context for understanding the distinct roles of ACSL isoforms discussed below. The following subsections focus on ACSL1, ACSL4, and ACSL6, for which disease-relevant evidence is available at different levels. By contrast, direct disease-specific evidence defining the functional roles of ACSL3 and ACSL5 in endometrial cancer remains insufficient, and these isoforms are therefore not discussed as separate mechanistic subsections.

### 6.1 ACSL1: the role in endometrial cancer

ACSL1 has been implicated in tumor-associated lipid metabolic reprogramming in several cancer models. For example, ACSL1 promotes the generation of fatty acyl-CoAs, mitochondrial fatty-acid β-oxidation, and ATP production in prostate cancer cells, whereas ACSL1 depletion suppresses cancer-cell proliferation, migration, and xenograft growth (107). ACSL1-dependent metabolic reprogramming has also been linked to enhanced metastatic behavior in ovarian cancer cells through altered fatty-acid metabolism and protein myristoylation (70). These findings provide tumor-cell-specific evidence that ACSL1 can couple fatty-acid activation to malignant phenotypes, although its effects remain cancer-type dependent. Obesity promotes the carcinogenic process in the endometrium through multiple pathways, including peripheral aromatization of estrogen, activation of insulin/IGF signaling pathways, and chronic inflammatory responses (108). Extending from these obesity-related pathways, substantial evidence indicates that ACSL1 is closely implicated in obesity-related metabolic dysregulation.

More directly, Zhou et al. demonstrated that ACSL1 is upregulated in human EC tissues and is associated with malignant progression. Functional experiments in EC cells showed that ACSL1 overexpression enhanced fatty-acid β-oxidation and ATP production and promoted cell proliferation and migration, whereas pharmacological inhibition of FAO partially attenuated these effects. Mechanistically, these changes were associated with activation of the AMPK/CPT1C/ATP axis (8). Together, these findings provide disease-specific evidence that ACSL1-dependent fatty-acid metabolism contributes to EC progression (**Figure 6**).

**Figure 6:**
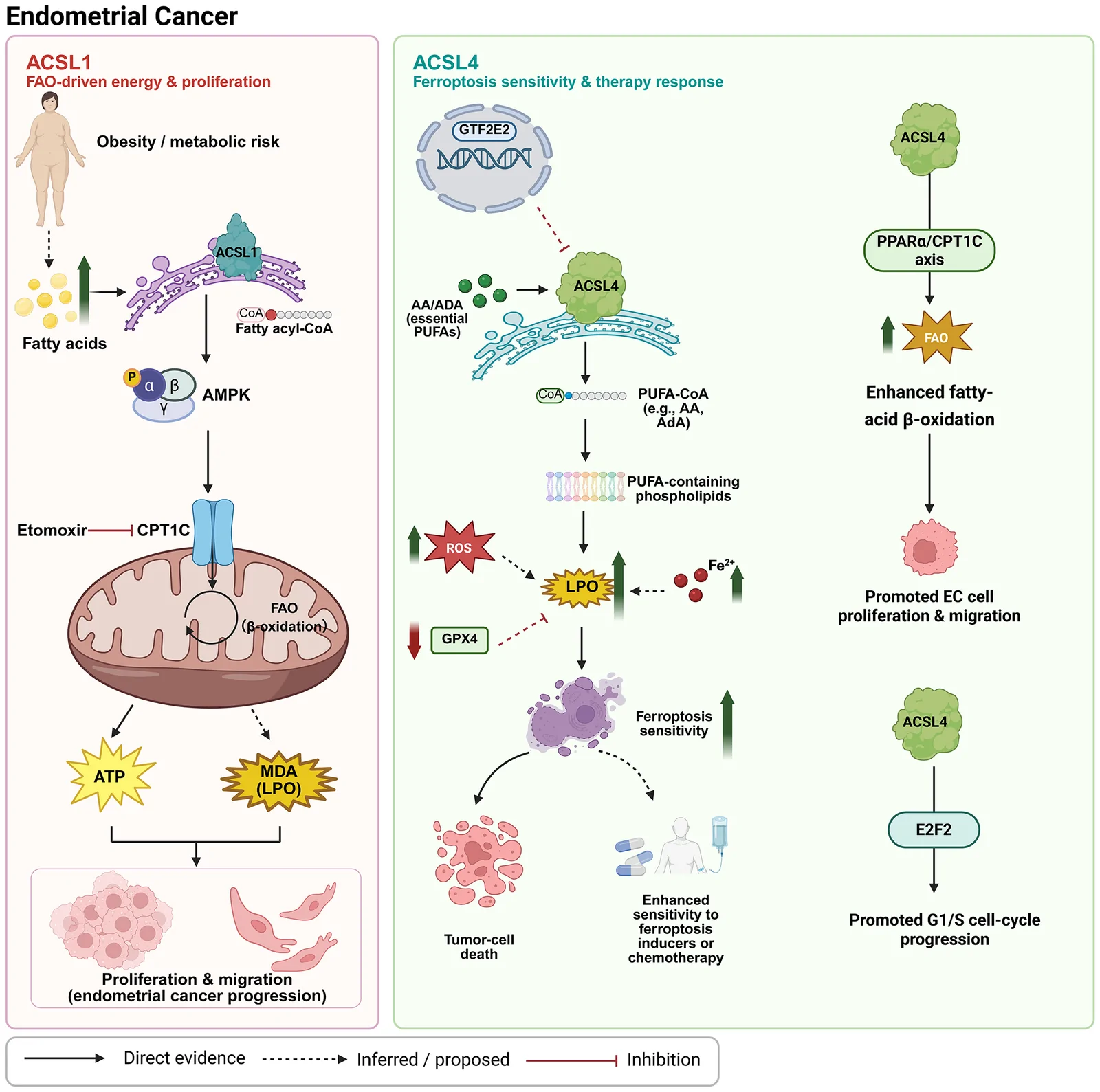
Isoform-specific roles of ACSL1 and ACSL4 in endometrial cancer. ACSL1 supports FAO through AMPK/CPT1C-associated signaling. ACSL4 promotes FAO and cell-cycle progression through PPARα/CPT1C and E2F2, respectively, whereas its causal contribution to GTF2E2-associated ferroptotic changes remains unresolved. Solid arrows indicate experimental support within EC models; dashed arrows indicate associative, inferred, or proposed relationships; red lines indicate inhibition. ACSL1, long-chain acyl-CoA synthetase 1; ACSL4, long-chain acyl-CoA synthetase 4; ACSL6, long-chain acyl-CoA synthetase 6; AMPK, adenosine monophosphate-activated protein kinase; ATP, adenosine triphosphate; CPT1C, carnitine palmitoyltransferase 1C; EC, endometrial cancer; FAO, fatty acid oxidation; Fe²^+^, ferrous iron; GTF2E2, general transcription factor IIE subunit 2; GPX4, glutathione peroxidase 4; LPO, lipid peroxidation; MUFA, monounsaturated fatty acid; ROS, reactive oxygen species; SCD, stearoyl-CoA desaturase; SREBP1c, sterol regulatory element-binding protein 1c; UCEC, uterine corpus endometrioid carcinoma.

### 6.2 ACSL4: the role in endometrial cancer

Disease-specific evidence increasingly supports a functional role for ACSL4 in EC. Guo et al. reported that ACSL4 is significantly upregulated in EC and inversely associated with tumor differentiation. Functional assays showed that ACSL4 promotes EC-cell proliferation, migration, and G1/S cell-cycle progression, whereas ACSL4 knockdown suppresses these phenotypes. Mechanistically, ACSL4 enhanced fatty-acid β-oxidation through the PPARα/CPT1C axis and promoted cell-cycle progression through E2F2; pharmacological ACSL4 inhibition also suppressed tumor growth *in vitro* and *in vivo (*109).

In addition, GTF2E2 knockdown in Uterine Corpus Endometrioid Carcinoma (UCEC) cells increased ACSL4 expression and was accompanied by increased intracellular Fe²^+^, lipid peroxides, and ROS and decreased GPX4 expression (110). GTF2E2 depletion also suppressed UCEC-cell proliferation, migration, and invasion. These findings support a GTF2E2–ACSL4-associated ferroptotic phenotype in EC cells; however, because the study primarily assessed ferroptosis-associated molecular and biochemical changes, additional ACSL4-specific rescue experiments would further establish the causal contribution of ACSL4 to ferroptotic cell death (110). More broadly, ferroptosis-related metabolic vulnerabilities have been described in EC, but their relationship to ACSL4 should be distinguished from direct ACSL4-specific functional evidence (111) (**Figure 6**).

### 6.3 ACSL6: the role in endometrial cancer

In contrast to ACSL1 and ACSL4, the role of ACSL6 in EC remains largely uncharacterized. Although EC tissues and cell models exhibit substantial alterations in fatty-acid composition, lipogenic enzyme expression, and SREBP-dependent lipid metabolism (106, 112), these studies do not directly implicate ACSL6. We therefore do not propose an ACSL6-specific metabolic mechanism in EC at present. Direct analyses of ACSL6 expression and functional perturbation in human EC tissues and EC cells will be required to determine whether this isoform contributes to EC-associated lipid remodeling (**Figure 6**).

## 7 ACSLs and preeclampsia

PE is a pregnancy-specific multisystem disorder characterized by new-onset hypertension, frequently accompanied by proteinuria and/or maternal organ dysfunction, and remains an important cause of maternal and fetal morbidity and mortality (113). Its pathophysiology involves abnormal placentation, placental stress, anti-angiogenic signaling, endothelial dysfunction, and systemic inflammatory responses (114, 115). Lipid-metabolic abnormalities are also associated with PE, with altered circulating triglycerides, free fatty acids, and lipoprotein profiles contributing to oxidative and vascular stress (114, 115). Increasing evidence further implicates placental lipid peroxidation and ferroptosis in trophoblast dysfunction and maternal vascular injury, providing a mechanistic context for examining ACSL isoforms in PE (116). The following discussion focuses on ACSL3, ACSL4, and ACSL5, while distinguishing direct disease-specific findings from associative or extrapolated evidence. Direct disease-specific evidence for ACSL1 and ACSL6 in preeclampsia remains insufficient, and these isoforms are therefore not discussed as separate mechanistic subsections.

### 7.1 ACSL3: the role in preeclampsia

Direct evidence linking ACSL3 to PE is currently lacking. PE is characterized by placental oxidative stress, lipid peroxidation, oxidized lipoproteins, and endothelial dysfunction (117–119), but these studies did not directly evaluate ACSL3. Given the established role of ACSL3 in fatty-acid activation and lipid partitioning, altered ACSL3 activity could theoretically influence placental lipid composition and susceptibility to lipid peroxidation; however, this remains a hypothesis and should not be considered an established PE mechanism. We therefore retain ACSL3 in the isoform-specific framework of this review as an explicitly unvalidated candidate (**Figure 7**), highlighting the need for direct expression and functional studies in human placental tissues and trophoblast models.

**Figure 7:**
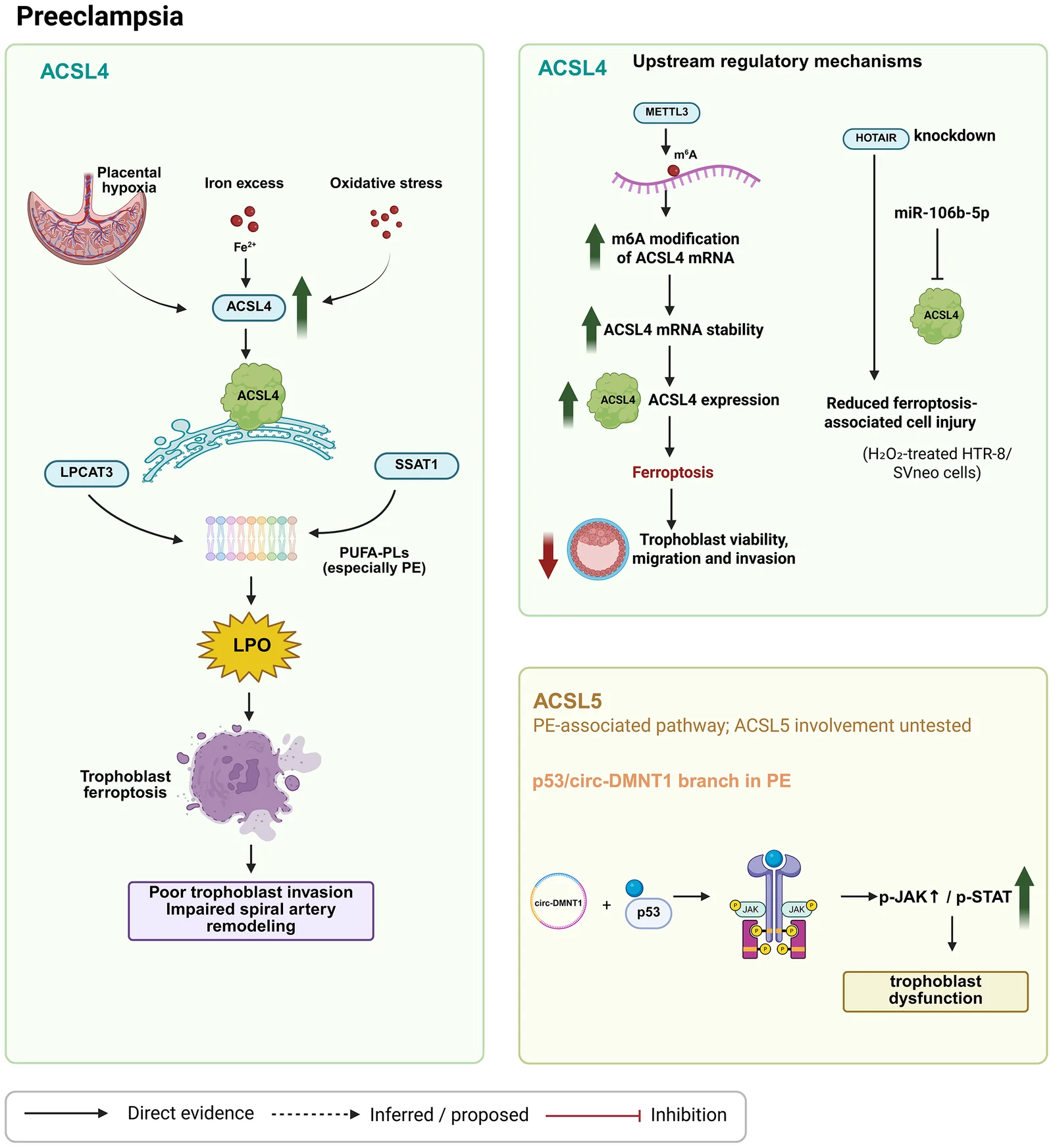
ACSL4-related ferroptosis and regulatory pathways in preeclampsia. METTL3 stabilizes ACSL4 mRNA through m6A modification, promoting trophoblast ferroptosis. HOTAIR/miR-106b-5p/ACSL4 signaling is implicated in oxidative-stress-induced trophoblast injury. The separate circ-DMNT1/p53/JAK/STAT3 pathway has no established ACSL5 involvement. Solid arrows indicate experimental support within specified models; dashed arrows indicate inferred or proposed relationships; red lines indicate inhibition. ACSL4, long-chain acyl-CoA synthetase 4; ACSL5, long-chain acyl-CoA synthetase 5; Fe²^+^, ferrous iron; H_2_O_2_, hydrogen peroxide; HOTAIR, HOX transcript antisense RNA; JAK, Janus kinase; LPO, lipid peroxidation; LPCAT3, lysophosphatidylcholine acyltransferase 3; m6A, N6-methyladenosine; METTL3, methyltransferase-like 3; miR, microRNA; mRNA, messenger RNA; p-, phosphorylated; PE, preeclampsia; PUFA-PLs, polyunsaturated fatty acid-containing phospholipids; SSAT1, spermidine/spermine N1-acetyltransferase 1; STAT3, signal transducer and activator of transcription 3.

### 7.2 ACSL4: the role in preeclampsia

Among the ACSL isoforms implicated in preeclampsia (PE), ACSL4 currently has the strongest disease-specific evidence. In placental tissues from women with late-onset PE (LO-PE), both ACSL4 mRNA and protein expression were significantly increased, particularly in the syncytiotrophoblast layer. These changes occurred together with increased expression of ferroptosis- and lipid-peroxidation-associated markers, including TFRC, ALOX5, GPX4, and MDA (47). However, because this clinical study primarily assessed molecular and histological markers, these findings should be interpreted as a ferroptosis-associated placental signature rather than definitive evidence that ACSL4 itself causes ferroptotic cell death *in vivo*.

Functional studies further support a role for ferroptosis in PE-associated trophoblast dysfunction. In a reduced uterine perfusion pressure rat model of PE, ferrostatin-1 attenuated ferroptosis-associated changes and increased fetal survival. In hypoxia-treated trophoblasts, manipulation of the miR-2115-3p/GOT1 axis altered cell proliferation and invasion together with ROS, ACSL4, TfR1, GPX4, SLC7A11, Fe²^+^, and MDA levels, supporting a link between hypoxia-induced ferroptosis and trophoblast dysfunction (120). Importantly, although ACSL4 changed in parallel with this pathway, the study did not demonstrate that miR-2115-3p or GOT1 directly regulates ACSL4; therefore, this pathway should be considered PE-specific ferroptosis evidence rather than an established upstream ACSL4 regulatory mechanism.

More direct evidence for ACSL4-dependent trophoblast injury has been provided by the METTL3/ACSL4 axis. METTL3 and ACSL4 were upregulated in placental tissues from patients with PE and in hypoxia-treated trophoblasts. METTL3-dependent m6A modification increased the stability of ACSL4 mRNA, whereas METTL3 silencing reduced ACSL4 expression, suppressed hypoxia-induced ferroptosis, and restored trophoblast viability, migration, and invasion. Importantly, ACSL4 re-expression partially reversed the protective effects of METTL3 silencing, providing functional rescue evidence that supports a causal contribution of the METTL3–ACSL4 axis to trophoblast ferroptosis. The effects of METTL3 were additionally evaluated in a PE rat model (121).

ACSL4 is also regulated by non-coding RNA signaling in trophoblast models. In H_2_O_2_-treated HTR-8/SVneo cells, HOTAIR knockdown alleviated ferroptosis-associated cell injury through the miR-106b-5p/ACSL4 axis, with ACSL4 identified as a downstream target of miR-106b-5p (122). Because this mechanism was established primarily in an oxidative-stress trophoblast model, further validation in PE placental tissues and *in vivo* models is required to determine its disease-level contribution. Collectively, current evidence identifies ACSL4 as an important interface between placental lipid-peroxidation susceptibility, hypoxic stress, and epigenetic/non-coding RNA regulation in PE (**Figure 7**). Nevertheless, therapeutic targeting of ACSL4 remains preclinical, and the efficacy and safety of ACSL4-directed intervention in PE have not yet been established.

### 7.3 ACSL5: the role in preeclampsia

Direct evidence linking ACSL5 to PE is currently lacking. PE-specific studies have demonstrated that the circ-DMNT1/p53/JAK/STAT3 axis can impair trophoblast viability, proliferation, migration, and invasion while promoting apoptosis (123), but ACSL5 was not evaluated in these experiments. Although ACSL5 can interact with stress- and lipid-metabolic pathways in other cellular contexts, extrapolating these findings to placental trophoblasts would be premature. Therefore, ACSL5 is retained here only as an unvalidated candidate within the isoform-specific framework (**Figure 7**); direct analysis of ACSL5 expression and functional perturbation in PE placentas and trophoblasts is required before assigning it a mechanistic role in PE.

## 8 Discussion and future perspectives

Across female reproductive diseases (FRDs), ACSL isoforms are best understood as context-dependent metabolic gatekeepers rather than uniformly pro- or anti-disease factors. Their biological effects depend on fatty-acid substrate preference, subcellular localization, cell type, and the metabolic pathway into which activated fatty acids are channeled (30, 31). ACSL4 illustrates this context dependence particularly clearly. In cervical cancer, ACSL4-dependent ferroptosis can suppress tumor growth and metastatic behavior (62), whereas in ovarian cancer, ACSL4-dependent PUFA-lipid synthesis can promote metastatic extravasation and colonization while simultaneously creating increased ferroptosis susceptibility (43). In non-neoplastic reproductive disorders, ACSL4-associated lipid peroxidation and ferroptotic phenotypes have been linked to impaired endometrial function in endometriosis (45, 97) and trophoblast dysfunction in preeclampsia (121, 122). These apparently divergent effects emphasize that the pathological consequences of ACSL4 activity cannot be inferred from isoform identity alone but must be interpreted according to disease and cellular context.

The strength of evidence also differs substantially among ACSL isoforms. ACSL4 currently has the broadest disease-specific functional support across the reproductive conditions reviewed, although the direction and mechanism of its effects remain disease- and cell-context dependent. ACSL1 has direct functional evidence in selected settings, particularly ovarian and endometrial cancer, but its roles cannot yet be generalized across FRDs. By contrast, many proposed functions of ACSL3 and ACSL5 remain based on transcriptomic associations, pathway-level observations, or mechanistic extrapolation from non-reproductive models, while ACSL6 remains poorly characterized in most reproductive diseases. Similarly, increases in ROS, MDA, Fe²^+^, or changes in GPX4 alone should be regarded as ferroptosis-associated evidence rather than definitive proof of ferroptotic cell death; stronger conclusions require ferroptosis-specific functional validation, ideally including rescue experiments and complementary genetic or mechanistic evidence. **Table 2** summarizes the supporting evidence, while **Figure 8** provides an overview of isoform-specific involvement and associated pathways across FRDs.

**Figure 8:**
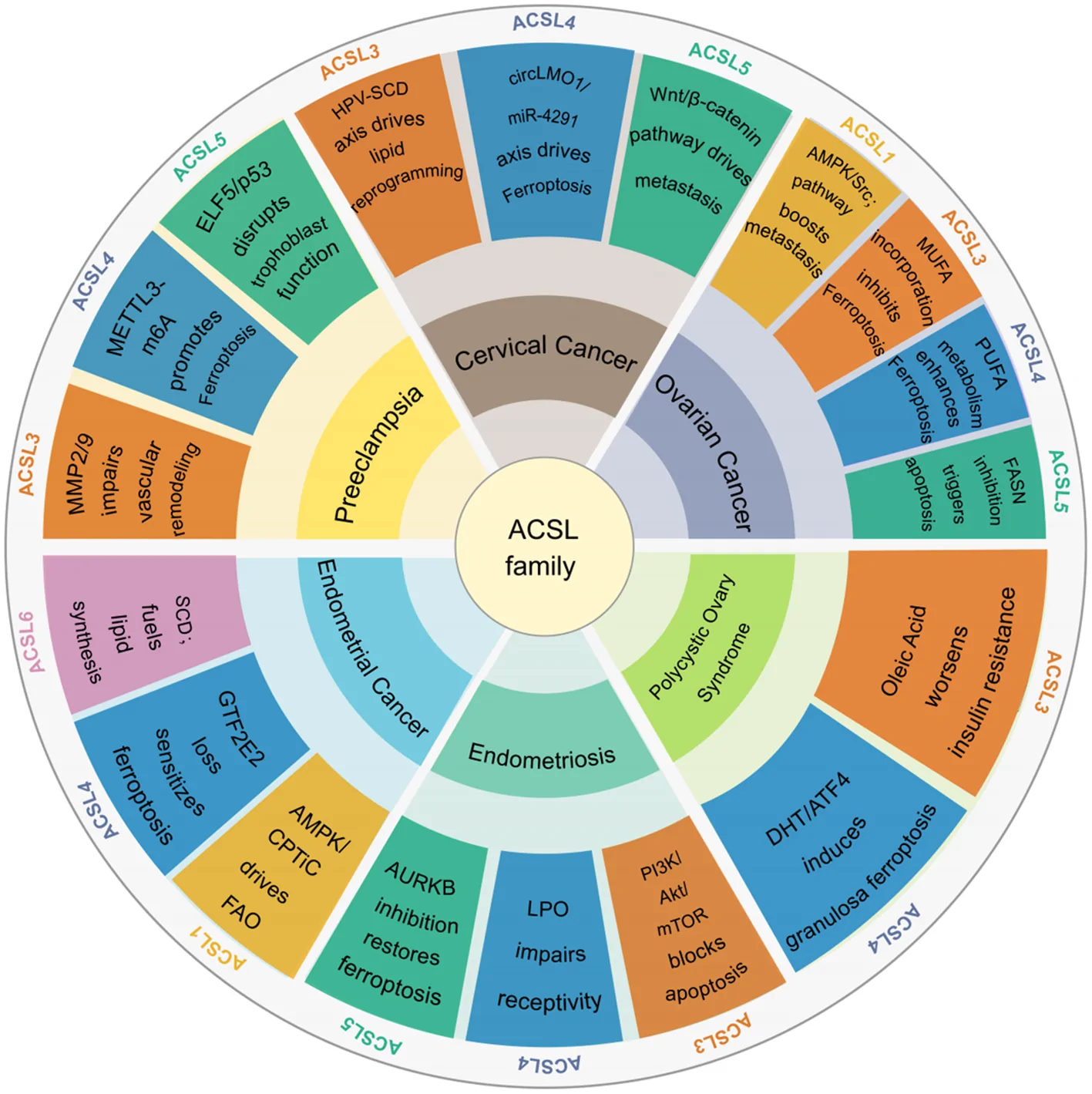
Isoform-specific involvement of ACSLs in female reproductive diseases. The circular diagram summarizes the associations between ACSL isoforms and disease-related pathways in female reproductive diseases. The center represents ACSLs, the inner sectors indicate disease categories, and the outer segments show specific ACSL isoforms and their associated mechanisms. These mechanisms include lipid reprogramming, ferroptosis, fatty acid oxidation, lipid synthesis, apoptosis, metastasis, insulin resistance, trophoblast dysfunction, vascular remodeling, and impaired endometrial receptivity. ACSL, long-chain acyl-CoA synthetase; FRDs, female reproductive diseases; HPV, human papillomavirus; SCD, stearoyl-CoA desaturase; PUFA, polyunsaturated fatty acid; MUFA, monounsaturated fatty acid; FASN, fatty acid synthase; LPO, lipid peroxidation; FAO, fatty acid oxidation; CPT1C, carnitine palmitoyltransferase 1C; SOD1, superoxide dismutase 1; GTF2E2, general transcription factor IIE subunit 2; AMPK, AMP-activated protein kinase; PI3K, phosphoinositide 3-kinase; Akt, protein kinase B; mTOR, mechanistic target of rapamycin; DHT, dihydrotestosterone; ATF4, activating transcription factor 4; MMP2/9, matrix metalloproteinase 2/9; METTL3, methyltransferase-like 3; m6A, N6-methyladenosine; ELF5, E74-like ETS transcription factor 5; p53, tumor protein p53.

Future studies should therefore prioritize causal, cell-type-resolved validation rather than further expansion of associative gene signatures. Single-cell and spatial transcriptomic approaches can identify the cellular sources of individual ACSL isoforms, whereas targeted lipidomics and stable-isotope tracing can determine how each isoform redirects fatty-acid flux into oxidation, storage, or oxidation-prone membrane phospholipids. These approaches should be combined with isoform-specific genetic perturbation in primary cells, patient-derived organoids, trophoblast models, and appropriate *in vivo* systems. Such integrated experiments will be particularly important for separating ACSL-dependent lipid remodeling from downstream ferroptosis-associated marker changes and for determining whether the same isoform exerts distinct effects across tumor, ovarian, endometrial, and placental cell populations.

Translation of ACSL biology into therapy also faces important pharmacological limitations. Selective pharmacological tools remain limited and are not broadly available across ACSL isoforms, although potent selective inhibitors have been developed for ACSL1 and, more recently, ACSL4 (124, 125). Earlier ACSL4-directed compounds also illustrate the importance of carefully evaluating target selectivity and off-target pharmacology; for example, rosiglitazone has ACSL4-inhibitory activity but also acts on PPARγ, whereas newer compounds have been developed to improve ACSL4 selectivity (125). Because ACSLs participate in physiological fatty-acid activation and channeling into distinct metabolic pathways (30), systemic inhibition may perturb normal lipid homeostasis outside the diseased tissue. Future therapeutic development should therefore emphasize isoform selectivity, tissue- or cell-targeted delivery, and pharmacodynamic biomarkers that confirm on-target lipid remodeling. These requirements are especially important in non-malignant reproductive disorders and pregnancy. Thus, the immediate priority is not broad clinical inhibition of the ACSL family, but rigorous validation of disease-specific ACSL dependencies followed by development of selective and context-appropriate intervention strategies.

## 9 Conclusion

In conclusion, ACSL isoforms provide an isoform- and context-specific link between lipid metabolic reprogramming, phospholipid remodeling, ferroptosis susceptibility, and inflammatory signaling in female reproductive diseases. Among the family members reviewed, ACSL4 currently has the strongest and most reproducible disease-specific functional evidence across multiple reproductive disorders, although its biological consequences differ among malignant and non-malignant tissues. ACSL1 has direct functional support in selected disease contexts, whereas ACSL3, ACSL5, and ACSL6 remain predominantly candidate metabolic nodes supported by more limited, associative, or extrapolated evidence. These knowledge gaps, together with the limited availability of isoform-selective pharmacological tools, currently preclude immediate clinical translation of most ACSL-directed strategies. Future progress will depend on cell-type-resolved functional studies, rigorous ferroptosis-specific validation, and the development of selective, tissue-appropriate approaches for modulating individual ACSL isoforms.

## Glossary

AA: arachidonic acid
ACSL: long-chain acyl-CoA synthetase
ACSL1: long-chain acyl-CoA synthetase 1
ACSL3: long-chain acyl-CoA synthetase 3
ACSL4: long-chain acyl-CoA synthetase 4
ACSL5: long-chain acyl-CoA synthetase 5
ACSL6: long-chain acyl-CoA synthetase 6
AKT: protein kinase B
ALOX5: arachidonate 5-lipoxygenase
AML: acute myeloid leukemia
AMPK: AMP-activated protein kinase
ATF4: activating transcription factor 4
ATP: adenosine triphosphate
AURKB: aurora kinase B
BCL2: B-cell lymphoma 2
BECN1: beclin 1
CC: cervical cancer
CD36: cluster of differentiation 36
CLA: conjugated linoleic acid
CPT1C: carnitine palmitoyltransferase 1C
DHA: docosahexaenoic acid
DHT: dihydrotestosterone
DNA: deoxyribonucleic acid
EC: endometrial cancer
ECH1: enoyl-CoA hydratase 1
ELF5: E74-like ETS transcription factor 5
EM: endometriosis
EPA: eicosapentaenoic acid
EPB41L1: erythrocyte membrane protein band 4.1-like 1
ER: endoplasmic reticulum
ET-1: endothelin-1
FABP4: fatty acid-binding protein 4
FAO: fatty acid oxidation
FASN: fatty acid synthase
Fe²⁺: ferrous iron
FRDs: female reproductive diseases
FSP1: ferroptosis suppressor protein 1
FZD7: frizzled class receptor 7
GEO: Gene Expression Omnibus
GPX4: glutathione peroxidase 4
GSH: glutathione
GTF2E2: general transcription factor IIE subunit 2
HDL: high-density lipoprotein
HM: highly metastatic
HNE: hydroxynonenal
HNF4A: hepatocyte nuclear factor 4 alpha
HOTAIR: HOX transcript antisense RNA
HPV: human papillomavirus
HUWE1: HECT, UBA and WWE domain-containing E3 ubiquitin protein ligase 1
ICGC: International Cancer Genome Consortium
IFN: interferon
IGF: insulin-like growth factor
IGF2BP3: insulin-like growth factor 2 mRNA-binding protein 3
IL6: interleukin 6
JAK: Janus kinase
JAK2: Janus kinase 2
JNK: c-Jun N-terminal kinase
LDL: low-density lipoprotein
LNM: lymph node metastasis
LPCAT3: lysophosphatidylcholine acyltransferase 3
LPO: lipid peroxidation
LXR: liver X receptor
m6A: N6-methyladenosine
MAPK: mitogen-activated protein kinase
MAPT: microtubule-associated protein tau
MBS: metabolic bariatric surgery
MDA: malondialdehyde
METTL3: methyltransferase-like 3
MMP2: matrix metalloproteinase 2
MMP9: matrix metalloproteinase 9
MUFA: monounsaturated fatty acid
NADPH: nicotinamide adenine dinucleotide phosphate
NM: non-metastatic
NO: nitric oxide
NOX1: NADPH oxidase 1
OC: ovarian cancer
oxLDL: oxidized low-density lipoprotein
PA: palmitic acid
PCOS: polycystic ovary syndrome
PCOS-IR: polycystic ovary syndrome with insulin resistance
PE: preeclampsia
PI3K: phosphoinositide 3-kinase
PINK1: PTEN-induced kinase 1
PLIN2: perilipin 2
PPAR: peroxisome proliferator-activated receptor
PRPF4B: pre-mRNA processing factor 4B
PTGS2: prostaglandin-endoperoxide synthase 2
PUFA: polyunsaturated fatty acid
PUFA-PE: polyunsaturated fatty acid-containing phosphatidylethanolamine
PUFA-PL: polyunsaturated fatty acid-containing phospholipid
RMS: rhabdomyosarcoma
RNA: ribonucleic acid
ROCK: Rho-associated coiled-coil-containing protein kinase
ROS: reactive oxygen species
SCD: stearoyl-CoA desaturase
SHBG: sex hormone-binding globulin
SLC7A11: solute carrier family 7 member 11
SNARE: soluble N-ethylmaleimide-sensitive factor attachment protein receptor
SOD1: superoxide dismutase 1
SOX2: SRY-box transcription factor 2
SP1: specificity protein 1
SREBP: sterol regulatory element-binding protein
SSAT1: spermidine/spermine-N1-acetyltransferase 1
STAT: signal transducer and activator of transcription
STAT3: signal transducer and activator of transcription 3
STX17: syntaxin 17
TCA: tricarboxylic acid
TCGA: The Cancer Genome Atlas
TFRC: transferrin receptor
TP53: tumor protein p53
TXNIP: thioredoxin-interacting protein
UCEC: uterine corpus endometrial carcinoma
VEGFA: vascular endothelial growth factor A
VIPAS39: VPS33B-interacting protein, apical-basolateral polarity regulator, spe-39 homolog
YBX1: Y-box binding protein 1.
